# Associations of (pre)diabetes with right ventricular and atrial structure and function: the Maastricht Study

**DOI:** 10.1186/s12933-020-01055-y

**Published:** 2020-06-15

**Authors:** Pauline B. C. Linssen, Marja G. J. Veugen, Ronald M. A. Henry, Carla J. H. van der Kallen, Abraham A. Kroon, Miranda T. Schram, Hans-Peter Brunner-La Rocca, Coen D. A. Stehouwer

**Affiliations:** 1 Cardiovascular Research Institute Maastricht, Maastricht University Medical Centre, Maastricht, The Netherlands; 2 Department of Internal Medicine, Maastricht University Medical Centre, Maastricht, The Netherlands; 3 Heart and Vascular Centre, Maastricht University Medical Centre, Maastricht, The Netherlands; 4 Department of Cardiology, Maastricht University Medical Centre, Maastricht, The Netherlands

**Keywords:** Heart failure, Echocardiography, Right atrial and ventricular structure, Right ventricular function, (pre)diabetes

## Abstract

**Backgrounds:**

The role of right ventricular (RV) and atrial (RA) structure and function, in the increased heart failure risk in (pre)diabetes is incompletely understood. The purpose of this study is to investigate the associations between (pre)diabetes and RV and RA structure and function, and whether these are mediated by left ventricular (LV) alterations or pulmonary pressure.

**Methods:**

Participants of the Maastricht Study; a population-based cohort study (426 normal glucose metabolism (NGM), 142 prediabetes, 224 diabetes), underwent two-dimensional and tissue Doppler echocardiography. Multiple linear regression analyses with pairwise comparisons of (pre)diabetes versus NGM, adjusted for cardiovascular risk factors, and mediation analyses were used.

**Results:**

In general, differences were small. Nevertheless, in individuals with prediabetes and diabetes compared to NGM; RA volume index was lower (both p < 0.01, p_trend_ < 0.01), RV diameter was lower (both p < 0.01, p_trend_ < 0.01) and RV length was significantly smaller in diabetes (p = 0.67 and p = 0.03 respectively, p_trend_ = 0.04), TDI S′RV was lower (p = 0.08 and p < 0.01 respectively, p_trend_ < 0.01), TDI E′RV was lower (p = 0.01 and p = 0.02 respectively, p_trend_ = 0.01) and TDI A′RV was lower (p < 0.01 and p = 0.07 respectively, p_trend_ = 0.04). Only the differences in RA volume index (7.8%) and RV diameter (6.2%) were mediated by the maximum tricuspid gradient, but no other LV structure and function measurements.

**Conclusions:**

(Pre)diabetes is associated with structural RA and RV changes, and impaired RV systolic and diastolic function, independent of cardiovascular risk factors. These associations were largely not mediated by indices of LV structure, LV function or pulmonary pressure. This suggests that (pre)diabetes affects RA and RV structure and function due to direct myocardial involvement.

## Background

The role of right ventricular (RV) structure and function in the increased risk of heart failure in patients with type 2 diabetes (T2DM) [1] and prediabetes [2] is incompletely understood [3–5] but may be important. Indeed, in a population free of overt cardiovascular disease [6], Kawut et al. showed that RV (independent of left ventricular (LV)) mass was associated with increased risk of heart failure. In another study [7], in heart failure patients, RV function was a stronger predictor of mortality than LV function.

In (pre)diabetes, parallel effects on RV and LV structure and function may occur through direct myocardial effects (e.g. fibrosis, oxidative stress, altered calcium homeostasis and substrate metabolism) [8–10]; through indirect effects via alterations in vascular function (e.g. arterial stiffening and microvascular dysfunction) [11]; and (or) through ventricular interdependence (i.e. structure, compliance, and function of the one ventricle, through direct mechanical interaction via septal wall and pericardium, may affect structure and function of the other ventricle) [3–5].

Associations of (pre)diabetes with RV structure and function have not been systematically investigated [9, 11–27]. Previous studies in general have been performed in selected and small populations (with type 1 diabetes [12, 14, 17, 25], in outpatient clinics [9, 12–17, 21–23, 25–27], with heart failure [24], with pulmonary hypertension [11], and (or) with less than 150 participants [12, 14–18, 20, 22, 24–27]), have not been adjusted for potential confounders [12, 14–17, 20, 22, 23, 25, 26], and have not investigated the role of prediabetes [11, 12, 14–18, 20–26]. In the population-based MESA study [19], (pre)diabetes was associated with lower RV mass and smaller RV volume, but not with RV systolic function, while RV diastolic function was not assessed.

In view of these considerations, we assessed associations between (pre)diabetes and RV and LV structure and function in the population-based Maastricht Study. In addition, we tested the hypothesis that RV alterations in (pre)diabetes are mediated by LV alterations and (or) pulmonary pressure.

## Methods

### Study population

We used data from The Maastricht Study, an observational prospective population-based cohort study. The rationale and methodology have been described previously [28]. In brief, the study focuses on the etiology, pathophysiology, complications and comorbidities of T2DM and is characterized by an extensive phenotyping approach. Eligible for participation were all individuals aged between 40 and 75 years and living in the southern part of the Netherlands. Participants were recruited through mass media campaigns and from the municipal registries and the regional Diabetes Patient Registry via mailings. Recruitment was stratified according to known T2DM status, with an oversampling of individuals with T2DM, for reasons of efficiency. The present report includes cross-sectional data from the first 866 participants, who completed the baseline survey between November 2010 and March 2012. To augment statistical power, another random sample of 218 participants was added, who had completed the baseline survey between April 2012 and April 2013. The examinations of each participant were performed within a time window of three months. Patients with type 1 diabetes mellitus or a paced heart rhythm were excluded. The study has been approved by the institutional medical ethical committee (NL31329.068.10) and the Minister of Health, Welfare and Sports of the Netherlands (Permit 131088-105234-PG). All participants gave written informed consent.

### Glucose metabolism status

To determine glucose metabolism status, all participants (except those who used insulin) underwent a standardized 2-h 75 g oral glucose tolerance test after an overnight fast. For safety reasons, participants with a fasting glucose level above 11.0 mmol/L, as determined by a finger prick, did not undergo the oral glucose tolerance test. For these individuals, fasting glucose level and information about diabetes medication were used to determine glucose metabolism status. Glucose metabolism status was defined according to the WHO 2006 criteria into normal glucose metabolism (NGM), impaired fasting glucose, impaired glucose tolerance (combined as prediabetes), and T2DM [29].

### Echocardiography

Echocardiograms were obtained according to a standardized protocol consisting of two-dimensional, M-mode, color flow Doppler, pulsed and continuous wave Doppler and Tissue Doppler recordings (TDI) with use of echo equipment (Vivid E9 with 2.5-3.5 MHz and 4 V transducer, GE Vingmed). All recordings were digitally stored and analyzed off-line (EchoPAC PC, version 112) by four researchers blinded to (pre)diabetes status. Details on echocardiographical procedures are provided in Additional file 1.

### Covariates

Office blood pressure (Omron 705IT, Omron, Japan) and ambulatory 24 h blood pressure (WatchBP O3, Microlife AG, Switzerland) were measured as described elsewhere [28]. Fasting serum concentrations of total cholesterol, high density lipoprotein (HDL) cholesterol, triglycerides and creatinine were measured (Beckman Synchron LX20, Beckman Coulter inc., Brea USA) [28]. Cystatin C was measured by a particle enhanced immunoturbidimetric assay (Roche Cobas 8000, Roche diagnostics, Basel, Switzerland). Estimated glomerular filtration rate (eGFR) was estimated according to the Chronic Kidney Disease Epidemiology Collaboration equation based on both serum creatinine and serum cystatin C [30]. Albuminuria, defined as an urinary albumin excretion ≥ 30 mg/24 h, was determined (twice) as described elsewhere [31]. Antihypertensive, lipid-modifying, and glucose-lowering medication use were assessed with a medication interview [28]. Renin-angiotensin system modifying agents were defined angiotensin converting enzyme inhibitors, angiotensin II inhibitors and (or) renin inhibitors use. Waist circumference was measured midway between the lower rib margin and the iliac crest end-expiratory. Alcohol consumption, smoking status (never, former, current), prevalent cardiovascular disease and physical activity were determined by questionnaire [28]. Alcohol consumption was categorized into non-consumers, low-consumers (≤ 7 and ≤ 14 glasses per week for females and males respectively) and high-consumers (> 7 and > 14 glasses per week for females and males respectively). Total and moderate to vigorous physical activity was assessed by a modified version of the Community Healthy Activities Model Program for Seniors (CHAMPS) questionnaire [32]. Prevalent cardiovascular disease was defined as a self-reported history of myocardial infarction, or cerebrovascular infarction or hemorrhage, or percutaneous artery angioplasty of, or vascular surgery of the coronary, abdominal, peripheral or carotid arteries. Other clinical characteristics (i.e. body mass index, waist-to-hip ratio, presence of hypertension, and HbA1c) were obtained from physical examination and laboratory assessment as described elsewhere [28].

### Statistical analyses

All analyses were performed with the statistical software package SPSS version 21.0 (SPSS IBM Corporation, Armonk, NY, USA). Descriptive statistics are presented as mean (± standard deviation), or in case of a skewed distribution as median [interquartile range] or frequencies (percentages). Variables with a skewed distribution were natural-log-transformed in order to meet normality criteria. Comparisons of population characteristics between groups were made by use of independent T-test or Chi squared test as appropriate. Dependent variables were standardized into sex specific Z-scores (individual value-mean_sex_)/(standard deviation_sex_). As sex had a skewed distribution in categories of glucose metabolism status, sex-specific means and standard deviations of the NGM group were used. Multiple linear regression analyses, both linear trend analyses and pairwise comparisons of prediabetes or T2DM versus NGM respectively (with dummy variables), were used to determine associations of (pre)diabetes with RA volume index, RV structure (i.e. RV diameter, RV length), and RV functions (i.e. TDI S’RV, TDI E’RV, TDI A’RV, TDI E′/A′ratio, RV myocardial performance index). Model 1 was adjusted for age; model 2 was additionally adjusted for cardiovascular risk factors associated with RV structure and functioning [19, 33] (i.e. systolic blood pressure, antihypertensive medication use, smoking status, prior cardiovascular disease, and waist circumference); and model 3 was additionally adjusted for albuminuria, eGFR, total to high density lipoprotein cholesterol ratio, triglycerides, and the use of lipid-modifying medication. Mediation analyses was performed to assess the extent to which ventricular interdependence and pulmonary hypertension statistically mediated the association between (pre)diabetes and RV structure and function. Hence, we added these factors (i.e. LV mass index, peak flow velocity E/longitudinal velocities E ratio, LV ejection fraction, maximum gradient of the tricuspid valve) to the aforementioned regression models. Both independent and joint mediation effects were expressed as the (relative) change of the regression coefficient. The corresponding 95% confidence intervals were assessed according to Preacher and Hayes (10,000 bootstrap iterations) [34]. Multicollinearity was assessed by collinearity diagnostics (i.e. tolerance < 0.1 and/or variance inflation factor > 10). A two-sided P-value < 0.05 was considered statistically significant, except for interaction analyses, where P < 0.10 was used.

## Results

### Study population

**Fig. 1:**
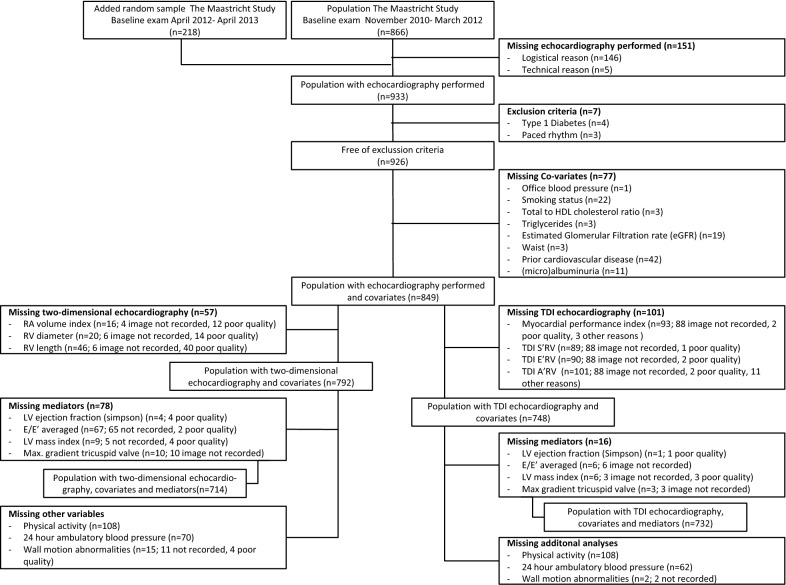
Selection of study population. *HDL* high density lipoprotein, *RA* right atrium, *RV* right ventricle, *2D* two-dimensional, *LV* left ventricle, *TDI* tissue Doppler imaging

Of the 1084 participants, echocardiography was obtained in 933 individuals. Participants were excluded due to exclusion criteria (n = 7), missing covariates (n = 72), and missing two-dimensional (n = 57) or TDI (n = 101) echocardiographic analyses, resulting in 792 and 748 participants eligible for current analyses respectively (Fig. 1). Individuals with missing data more frequently had T2DM; however, within strata of (pre)diabetes, these individuals were largely comparable (Additional file 2: Table S1).

### Characteristics

Characteristics of the total study population and stratified according to (pre)diabetes are given in Tables 1, 2 and Additional file 2: Table S2. Participants with (pre)diabetes had a worse cardiovascular risk profile; they were older and more often men, had a higher body mass index, greater waist circumference, higher blood pressure, lower HDL, higher triglycerides, lower eGFR, and less physical activity. They also more frequently had prior cardiovascular disease, hypertension and albuminuria, and more often used antihypertensive and lipid-modifying medication (Table 1, Additional file 2: Table S2).

**Table 1:** Clinical characteristics of the study population with two-dimensional echocardiography according to glucose metabolism status (n = 792)

Variable	NGM (n = 426)	Prediabetes (n = 142)	T2DM (n = 224)
Demographics			
Age, years	57.3 ± 8.0	62.5 ± 7.5^‡^	62.6 ± 7.6^‡^
Women, %	233 (54.7)	57 (40.1)^‡^	71 (31.7)^‡^
Measures of (central) obesity			
Body mass index, kg/m^2^	25.5 [23.2–27.8]	27.4 [25.6–29.8]^‡^	29.3 [26.2–32.2]^‡^
Waist circumference, cm			
Men	96 [91–103]	102 [96–110]^‡^	106 [99–113]^‡^
Women	87 [79–94]	94 [85–100]^‡^	101 [91–113]^‡^
Waist-to hip ratio^a^	0.91 ± 0.08	0.97 ± 0.08^‡^	1.01 ± 0.08^‡^
Blood pressure			
Office systolic blood pressure, mmHg	131.1 ± 16.5	140.0 ± 17.2^‡^	145.0 ± 18.3^‡^
Office diastolic blood pressure, mmHg	75.2 ± 9.7	79.3 ± 10.3^‡^	78.0 ± 9.3^‡^
24 h average ambulatory systolic blood pressure, mmHg^b^	116.3 ± 10.6	121.6 ± 12.8^‡^	122.1 ± 11.5^‡^
24 h average ambulatory diastolic blood pressure, mmHg^b^	73.6 ± 7.0	74.8 ± 8.0	73.4 ± 7.1
Hypertension, %	169 (39.7)	98 (69.0)^‡^	187 (83.5)^‡^
Glucose metabolism			
Fasting glucose, mmol/L	5.2 ± 0.4	6.0 ± 0.5**	7.9 ± 2.3**
2 h postload glucose, mmol/L^c^	5.3 ± 1.2	8.2 ± 1.8**	14.0 ± 3.4**
HbA1c, mmol/mol^d^	37.1 ± 3.5	40.4 ± 4.2**	51.7 ± 11.2**
HbA1c, %^d^	5.5 ± 0.3	5.8 ± 0.4**	6.9 ± 1.0**
Lipids			
Total cholesterol, mmol/L	5.6 ± 1.0	5.5 ± 1.2	4.5 ± 1.0^‡^
HDL cholesterol, mmol/L			
Men	1.3 ± 0.3	1.2 ± 0.3	1.1 ± 0.3^‡^
Women	1.7 ± 0.5	1.6 ± 0.4	1.4 ± 0.4^‡^
Total to HDL cholesterol ratio	4.0 ± 1.3	4.3 ± 1.3*	4.0 ± 1.1
LDL cholesterol, mmol/l	3.6 ± 0.9	3.4 ± 1.0	2.5 ± 0.9^‡^
Triglycerides, mmol/l	1.0 [0.8–1.5]	1.4 [0.9–1.9]^‡^	1.7 [1.2–2.3]^‡^
Kidney function			
eGFR, ml/min/1.73 m^2^	90.8 ± 13.8	85.0 ± 14.4^‡^	85.2 ± 17.2^‡^
Albuminuria, %	15 (3.5)	10 (7.0)*	38 (17.0)^‡^
Lifestyle			
Smoking (never, former, current), %	153/198/75 (35.9/46.5/17.6)	41/85/16 (28.9/59.9/11.3)*	59/128/37 (26.3/57.1/16.5)^†^
Alcohol consumption (none, low, high), %^e^	52/226/145 (12.3/53.4/34.3)	21/70/51 (14.8/49.3/35.9)	66/109/48 (29.6/48.9/21.5)^‡^
Moderate to vigorous physical activity, h/wk^f^	5.3 [3.0–8.5]	4.5 [2.0–6.8]^‡^	3.8 [1.6–6.5]^‡^
Prior cardiovascular disease, %	44 (10.3)	23 (16.2)*	56 (25.0)^‡^
Medication			
Antihypertensive medication use, %	97 (22.8)	68 (47.9)^‡^	154 (68.8)^‡^
Ras inhibitors, %	64 (15.0)	49 (34.5)^‡^	124 (55.4)^‡^
Beta-blockers, %	36 (8.5)	32 (22.5)^‡^	81 (36.2)^‡^
Diuretics, %	29 (6.8)	29 (20.4)^‡^	60 (26.8)^‡^
Calcium antagonists, %	15 (3.5)	11 (7.7)^†^	35 (15.6)^‡^
Diabetes medication use, %	–	–	166 (74.1)**
Insulin, %	–	–	40 (17.9)**
Metformin, %	–	–	151 (67.4)**
Sulfonylureas, %	–	–	41 (18.3)**
Thiazolidinediones, %	–	–	2 (0.9)**
GLP-1 analogs, %	–	–	2 (0.9)**
DPP-4 inhibitors, %	–	–	5 (2.2)**
Lipid modifying medication use, %	68 (16.0)	54 (38.0)^‡^	166 (74.1)^‡^

Data are presented as n (%), mean ± standard deviation or median (interquartile rage)

*NGM* normal glucose metabolism, *T2DM* type 2 diabetes, *HDL* high density lipoprotein, *LDL* low density lipoprotein, *eGFR* estimated glomerular filtration rate, *Ras* renin-angiotensin system, *GLP-1* glucagon-like peptide-1, *DPP-4* dipeptidyl peptidase-4

Numbers of missing data: ^a^ n = 1, ^b^ n = 70, ^c^ n = 52, ^d^ n = 3, ^e^ n = 4, ^f^ n = 108

P value difference prediabetes or T2DM vs NGM: * < 0.10, ^†^ < 0.05, ^‡^ < 0.01, ** not applicable

Participants with (pre)diabetes had lower LV end diastolic volume index, higher LVMI, worse LV systolic function, and worse LV diastolic function (lower E/A ratio, higher E/E′-ratio averaged, more frequent abnormal diastolic function grade). In addition, they had lower RA volumes, worse RV systolic function, and worse RV diastolic function (lower E/A ratio of the tricuspid valve, lower E′RV). Participants with (pre)diabetes more often had wall motion abnormalities (Table 2).

**Table 2:** Echocardiographic characteristics of the study population according to glucose metabolism status

Variable	NGM (n = 426)		Prediabetes (n = 142)		T2DM (n = 224)	
Left atrium						
LA volume index, ml/m^2^						
Men	30.8 [26.6–34.9]		28.9 [25.0–34.7]		29.7 [25.2–34.5]	
Women	28.6 [24.7–33.3]		28.6 [25.3–33.2]		26.3 [23.2–32.4]	
LA volume, ml						
Men	62.0 [53.3–72.4]		62.3 [50.9–72.9]		61.1 [52.4–72.0]	
Women	50.9 [43.6–59.2]		52.9 [43.6–59.2]		51.3 [42.3–59.6]	
Left ventricle, structure						
LV end diastolic volume index, ml/m^2^						
Men	68.5 ± 12.5		65.9 ± 11.8		63.0 ± 12.3^‡^	
Women	59.1 ± 10.6		58.1 ± 11.3		57.6 ± 11.9	
LV end systolic volume index, ml/m^2^						
Men	27.5 ± 6.1		26.7 ± 6.2		25.9 ± 6.3^†^	
Women	23.2 ± 4.6		23.0 ± 5.0		22.8 ± 5.3	
LV mass index, gr/m^2.7^						
Men	29.5 [25.3–35.1]		31.5 [27.4–35.0]		32.8 [27.0–37.5]^‡^	
Women	27.3 [24.0–31.3]		31.1 [25.5–36.7]^‡^		31.2 [27.1–34.7]^‡^	
Left ventricular function						
Systolic LV function						
LV ejection fraction, %	60.5 [58.8–62.4]		60.4 [58.8–62.0]		60.2 [57.8–61.7]^‡^	
TDI S’ septal (cm/s)^a^	7.5 ± 1.4		7.3 ± 1.3		7.4 ± 1.7	
TDI S’ lateral (cm/s)^b^	8.8 ± 2.0		8.8 ± 2.1		8.2 ± 2.0^‡^	
Diastolic LV function						
Early peak velocity (m/s)	0.68 ± 0.15		0.66 ± 0.14		0.68 ± 0.15	
Active peak velocity (m/s)^c^	0.66 ± 0.15		0.72 ± 0.17^‡^		0.75 ± 0.16^‡^	
E/A ratio^c^	1.03 [0.85–1.26]		0.89 [0.75–1.08]^‡^		0.89 [0.77–1.07]^‡^	
Deceleration time E-peak (msec)	191 ± 35		204 ± 38^‡^		202 ± 37^‡^	
Isovolumetric relaxation time (msec)^d^	94 ± 21		100 ± 24^‡^		94 ± 22	
S/D ratio^e^	1.38 ± 0.32		1.45 ± 0.36^†^		1.46 ± 0.31^‡^	
TDI E’ septal (cm/s)^b^	8.3	± 2.2	7.1	± 1.7^‡^	7.1	± 1.8^‡^
TDI A’ septal (cm/s)^f^	9.8 ± 1.8		9.8 ± 1.7		10.0 ± 1.9	
TDI E’ lateral (cm/s)^g^	10.7 ± 2.7		9.5 ± 2.5^‡^		8.9 ± 2.2^‡^	
TDI A’ lateral (cm/s)^h^	10.6 ± 2.5		11.1 ± 2.4^†^		11.1 ± 2.3^†^	
E/E’-ratio averaged^i^	7.3 [6.2–8.7]		8.0 [6.9–9.7]^‡^		8.7 [7.6–10.1]^‡^	
Diastolic LV function (normal, indeterminate, abnormal, not specified), n (%)	175/183/42/26 (41.1/43.0/9.9/6.1)		30/79/23/10 (21.1/55.6/16.2/7.0)^‡^		44/115/40/25 (19.6/51.3/17.9/11.2)^‡^	
Right atrium						
RA volume index, ml/m^2^						
Men	25.2 ± 7.2		22.1 ± 6.8^‡^		21.7 ± 6.2^‡^	
Women	21.3 ± 6.0		19.7 ± 5.5		18.3 ± 5.3^‡^	
RA volume, ml						
Men	48.1 [40.0–58.8]		43.4 [35.3–54.8]^‡^		43.7 [36.7–51.9]^‡^	
Women	36.0 29.7–45.1		36.0 [28.6–40.7]		33.7 [28.0–40.2]^†^	
Right ventricle, structure						
RV diameter						
Men	41.3 ± 5.2		39.9 ± 5.3^†^		39.8 ± 4.7^‡^	
Women	35.6 ± 4.7		35.8 ± 5.6		35.0 ± 4.2	
RV length						
Men	75.8 ± 7.5		75.8 ± 7.0		75.9 ± 7.2	
Women	69.8 ± 6.6		70.3 ± 6.2		70.2 ± 7.3	
Right ventricular function						
Systolic RV function						
S’ RV (cm/s)^j^	12.8 ± 2.4		12.4 ± 2.2		12.4 ± 2.5	
TAPSE (mm)^k^	23.0 ± 2.9		22.0 ± 3.6		22.3 ± 4.4	
Diastolic RV function						
E-peak velocity tricuspid flow (m/s)^l^	0.46 ± 0.09		0.44 ± 0.09		0.44 ± 0.08	
A-peak velocity tricuspid flow (m/s)^m^	0.35 ± 0.10		0.41 ± 0.07^†^		0.41 ± 0.09^‡^	
E/A ratio tricuspid valve^m^	1.30 [1.12–1.58]		1.10 [0.91–1.22]^‡^		1.10 [0.90–1.26]^‡^	
TDI E’ RV (cm/s)^n^	11.9 ± 2.7		10.4 ± 2.7^‡^		10.5 ± 2.7^‡^	
TDI A’ RV (cm/s)^o^	14.6 ± 3.5		13.9 ± 3.1^†^		14.1 ± 3.4	
E/E′-ratio^k^	4.3 [3.6–5.0]		4.8 [4.1–5.7]		4.6 [3.4–5.9]	
Global RV function						
Myocardial performance index RV^p^	0.47 ± 0.12		0.47 ± 0.12		0.48 ± 0.11	
Maximum tricuspid gradient	15.8 ± 6.2		14.7 ± 7.6		14.9 ± 7.2	
Wall motion abnormalities, n yes (%)^q^	2 (0.5)		2 (1.4)		5 (2.3)^†^	
Valvular dysfunction (moderate or severe), n (%)	28 (6.6)		8 (5.6)		13 (5.8)	

Data are presented as n (%), mean ± standard deviation or median (interquartile range)

*LA* left atrial, *LV* left ventricular, *TDI* tissue Doppler imaging, *RA* right atrial, tricuspid annular plane systolic excursion, *RV* right ventricular

Numbers of missing data (if n > 10); ^a^ n = 62; ^b^ n = 63; ^c^ n = 14; ^d^ n = 26; ^e^ n = 22; ^f^ n = 72; ^g^ n = 64; ^h^ n = 73; ^i^ n = 67; ^j^ n = 70; ^k^ n = 645; ^l^ n = 644; ^m^ n = 647; ^n^ n = 71; ^o^ n = 80; ^p^ n = 74; ^q^ n = 15

P value difference prediabetes or T2DM vs NGM: * < 0.10, ^†^ < 0.05, ^‡^ < 0.01

### Associations between (pre)diabetes and RA and RV structure and function

RA volume index was lower in individuals with prediabetes and T2DM compared to NGM (p < 0.01 and p < 0.01 respectively, p for trend < 0.01; Table 3). Adjustment for potential confounders of models 2 and 3 attenuated the difference in RA volume index, although the association remained statistically significant (p < 0.01 and p < 0.01 respectively, p for trend < 0.01).

**Table 3:** Multivariable adjusted differences of right ventricle structure and function between individuals with (pre)diabetes, as compared to individuals with normal glucose metabolism

Variable	Model	NGM	Prediabetes	T2DM	P for trend
			B (95% CI)	B (95% CI)	
Right atrium					
RA volume index (SD)^a^	1	Ref	− 0.39 (− 0.58; − 0.21)^‡^	− 0.52 (− 0.68; − 0.36)^‡^	< 0.01
	2	Ref	− 0.32 (− 0.51; − 0.13)^‡^	− 0.39 (− 0.57; − 0.21)^‡^	< 0.01
	3	Ref	− 0.26 (− 0.45; − 0.07)^‡^	− 0.29 (− 0.48; − 0.09)^‡^	< 0.01
Right ventricle, structure					
RV diameter (SD)^a^	1	Ref	− 0.12 (− 0.31; 0.08)	− 0.20 (− 0.37; − 0.04)^†^	0.02
	2	Ref	− 0.29 (− 0.49; − 0.09)^‡^	− 0.49 (− 0.68; − 0.30)^‡^	< 0.01
	3	Ref	− 0.27 (− 0.47; − 0.07)^‡^	− 0.44 (− 0.65; − 0.24)^‡^	< 0.01
RV length (SD)^a^	1	Ref	0.15 (− 0.04; 0.34)	0.15 (− 0.01; 0.32) *	0.06
	2	Ref	− 0.02 (− 0.22; 0.17)	− 0.15 (− 0.33; 0.04)	0.13
	3	Ref	− 0.04 (− 0.24; 0.15)	− 0.22 (− 0.42; − 0.02)^†^	0.04
Systolic RV function					
S’ RV (SD)^b^	1	Ref	− 0.18 (− 0.37; 0.02) *	− 0.26 (− 0.44; − 0.09)^‡^	< 0.01
	2	Ref	− 0.21 (− 0.41; 0.00)^†^	− 0.30 (− 0.50; − 0.10)^‡^	< 0.01
	3	Ref	− 0.19 (− 0.39; 0.02) *	− 0.29 (− 0.51; − 0.07)^‡^	0.01
Diastolic RV function					
TDI E’ RV (SD)^b^	1	Ref	− 0.31 (− 0.50; − 0.11)^‡^	− 0.36 (− 0.53; − 0.18)^‡^	< 0.01
	2	Ref	− 0.30 (− 0.50; − 0.10)^‡^	− 0.31 (− 0.51; − 0.11)^‡^	< 0.01
	3	Ref	− 0.26 (− 0.47; − 0.06)^†^	− 0.26 (− 0.48; − 0.05)^†^	0.01
TDI A’ RV (SD)^b^	1	Ref	− 0.32 (− 0.51; − 0.13)^‡^	− 0.29 (− 0.46; − 0.12)^‡^	< 0.01
	2	Ref	− 0.29 (− 0.49; − 0.10)^‡^	− 0.23 (− 0.42; − 0.10)^‡^	0.01
	3	Ref	− 0.26 (− 0.46; − 0.07)^‡^	− 0.20 (− 0.41; 0.01) *	0.04
TDI E′/A′ ratio (SD)^b^	1	Ref	− 0.02 (− 0.19; 0.15)	− 0.07 (− 0.22; 0.08)	0.39
	2	Ref	− 0.03 (− 0.20; 0.15)	− 0.08 (− 0.25; 0.09)	0.35
	3	Ref	− 0.02 (− 0.19; 0.16)	− 0.07 (− 0.25; 0.12)	0.50
Global RV function					
Myocardial performance index RV (SD)^b^	1	Ref	− 0.04 (− 0.23; 0.16)	− 0.04 (− 0.21; 0.13)	0.63
	2	Ref	− 0.06 (− 0.26; 0.15)	− 0.09 (− 0.29; 0.12)	0.40
	3	Ref	− 0.10 (− 0.31; 0.11)	− 0.17 (− 0.39; 0.05)	0.12

Model 1: adjusted for age. Model 2: adjusted for model 1 + office systolic blood pressure, antihypertensive medication, smoking status, prior cardiovascular disease, waist circumference. Model 3: adjusted for model 2 + albuminuria, eGFR, total to high density lipoprotein cholesterol ratio, triglycerides, the use of lipid-modifying medication

*CI* confidence interval, *NGM* normal glucose metabolism, RA right atrial, *RV* right ventricular, *SD* standard deviation, *T2DM* type 2 diabetes mellitus, *TDI* tissue Doppler imaging

Study population ^a^ n = 792, ^b^ n = 748

P value difference prediabetes or T2DM vs NGM: * < 0.10, ^†^ < 0.05,^‡^ < 0.01

RV diameter was lower in individuals with prediabetes and T2DM compared to NGM (p = 0.24 and p = 0.02 respectively, p for trend 0.02; Table 3). Adjustment for potential confounders of models 2 and 3 strengthened the difference in RV diameter (p < 0.01 and p < 0.01 respectively, p for trend < 0.01). RV length was not significantly different in individuals with prediabetes and T2DM compared to NGM (p = 0.12 and p = 0.07, p for trend 0.06); however, after adjustment for potential confounders, RV length was not different in prediabetes but significantly smaller in T2DM compared to NGM (p = 0.67 and p = 0.03 respectively, p for trend = 0.04).

TDI S′RV was lower in individuals with prediabetes and T2DM compared to NGM (p = 0.08 and p < 0.01 respectively, p for trend < 0.01; Table 3). Adjustment for potential confounders had no effect on this difference.

Both TDI E′RV and A′RV were lower in individuals with prediabetes and T2DM compared to NGM (for both measurements p < 0.01 and p < 0.01 respectively, p for trend < 0.01; Table 3). Adjustment for potential confounders attenuated these differences, although the association remained significant in TDI E′RV (p = 0.01 and p = 0.02 respectively, p for trend = 0.01) and (borderline) significant in A′RV (p < 0.01 and p = 0.07 respectively, p for trend = 0.04). TDI RV E′/A′ ratio was not significantly different in individuals with prediabetes and T2DM compared to NGM (p = 0.84 and p = 0.38 respectively, p for trend = 0.39), nor after full adjustment (p = 0.85 and p = 0.49 respectively, p for trend 0.50).

The RV myocardial performance index was not significantly different in individuals with prediabetes and T2DM compared to NGM (p = 0.71 and p = 0.65 respectively, p for trend = 0.63; Table 3), nor after full adjustment (p = 0.34 and p = 0.12 respectively, p for trend 0.12).

### Mediation

**Fig. 2:**
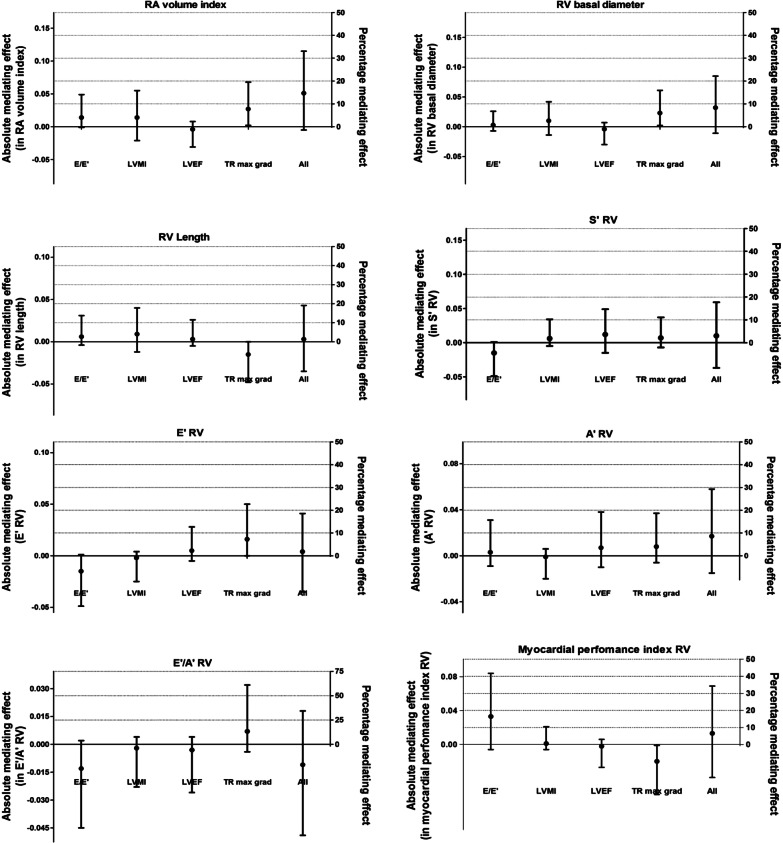
Mediating effects. Mediating effects are presented as indirect effects of T2DM on RV structure and function (absolute effect on left Y-axis, relative effect in percentages on right Y-axis) through potential mediators. All analyses are adjusted for age, office systolic blood pressure, antihypertensive medication, smoking status, prior cardiovascular disease, waist circumference albuminuria, eGFR, total to high density lipoprotein cholesterol ratio, triglycerides, the use of lipid-modifying medication. *RV* right ventricle, *RA* right atrium, *LVMI* left ventricular mass index, *E/E*′ peak flow velocity E/longitudinal velocities E ratio, *LVEF* left ventricular ejection fraction, *TR* max grad maximum gradient of the tricuspid valve

The difference in RA volume index between individuals with T2DM and those with NGM was partly mediated by the maximum gradient of the tricuspid valve (statistical mediating effect 7.8%, bootstrapped 95% CI 0.1–20.1%; Fig. 2); E/E′, LVMI, or LVEF had no significant mediating effects. The difference in RV diameter between individuals with T2DM and those with NGM was partly mediated by the maximum gradient of the tricuspid valve (statistical mediating effect 6.2%, bootstrapped 95% CI 0.3–16.4%); E/E′, LVMI, or LVEF had no significant mediating effects. Differences in RV length, S′RV, E′RV, or A′RV between individuals with T2DM and those with NGM were not significantly mediated by the maximum gradient of the tricuspid valve, E/E′, LVMI, or LVEF (Additional file 2: Table S3a, b).

### Additional analyses

In the associations of (pre)diates with RV and RA structure and function, no interaction between (pre)diabetes and sex was observed. Furthermore, in these associations no interaction between (pre)diabetes and age was observed except for the RV E′/A′ (p-interaction = 0.01); the difference between individuals with T2DM and NGM was greater in the youngest compared to the oldest; however, both differences were not statistically significant (data not shown). The results of statistical analyses did not materially change when these were repeated on those with full echocardiographic data (mediators included); when office systolic blood pressure was replaced by ambulatory 24-h systolic blood pressure; when analyses were additionally adjusted for renin-angiotensin system modifying agents or moderate to vigorous physical activity; or subjects were excluded with atrial fibrillation, wall motion abnormalities, significant valvular pathology, and prior cardiovascular disease (Additional file 2: Table S4a–e).

## Discussion

This study shows that both prediabetes and T2DM are associated with structural RA and RV changes, and impaired RV systolic and diastolic function, and that these associations are independent of other traditional cardiovascular risk factors. The associations between (pre)diabetes and RA and RV structure and function were largely not statistically mediated by indices of LV structure, LV function or pulmonary pressure, except for the associations between (pre)diabetes and RA and RV structure, which were statistically mediated by pulmonary pressure to a very limited extent.

This study extends previous research because of the assessment of RA and RV structure and RV function (i.e. systolic and diastolic) in a relatively large population-based study, with special emphasis on prediabetes; the comprehensive clinical characterization, which enables extensive adjustment for potential confounders; and statistical mediation analyses to investigate the role of LV structure and function in these associations. With regard to the RV structure, and in agreement with earlier studies [9, 18, 19] we showed that pre(diabetes) was associated with a smaller RV (i.e. RV diameter and RV length) although some smaller and(or) unadjusted studies [12–14, 16, 17, 22, 24–27] did not find this association. With regard to RV systolic function, and in agreement with earlier study [24–27], we showed that (pre)diabetes is associated with RV impaired systolic function independent of traditional risk factors, in contrast to most smaller and(or) unadjusted studies [9, 11–14, 16–18, 20, 22], which found no association. The Mesa study [19], which used RV ejection fraction as measurement for systolic function, found no association either. This could be explained by the fact that TDI-derived measurements, which are used in our study, are more sensitive to alteration of RV systolic function [35]. With regard to diastolic function, in agreement with most earlier studies [9, 13, 14, 17, 18, 20, 22, 24–27] we showed that (pre)diabetes was associated with impaired RV diastolic function (lower E′RV, lower E/A ratio of the tricuspid valve). In contrast to other studies [14, 17, 22, 25–27] we observed a lower A′RV in (pre)diabetes, although the latter studies were unadjusted [14, 17, 22, 25–27] and(or) performed in a selected study population (i.e. normotensive, younger, and (or) with type 1 diabetes patients) [14, 17, 25].

### Pathophysiology

Although in (pre)diabetes parallel effects on RV and LV structure and function may occur, our results show that the associations between (pre)diabetes and RA and RV structure and function were not mediated by indices of LV structure of function. Since there was no collinearity in mediation analyses, these results suggest a direct myocardial effect, which may apply to both ventricles independently of each other. For instance, as investigated in the RV of Zucker diabetic fatty rats versus controls, lower metabolic rates of glucose utilization and reduced insulin sensitivity were observed [36], which parallels the metabolic changes of the LV in these rats [37]. The subsequent hyperglycemia may alter calciumhomeostasis by inducing calcium/calmodulin-depent kinase II-delta activity as shown in RA and RV myocardium of patients with T2DM and Zucker diabetic fatty rats, which was associated with altered cardiac contractility and relaxation [10]. Nevertheless, Song et al. did show that the expression of cardiac metabolism (i.e. AKT activity and glucose transporter 4) differs among the RV, LV, and interventricular septum [38]. As the RV has, in comparison to the LV, lower mass (i.e. less oxygen consumption), a higher compliance, and lower pressures [4], it is possible that the similar pathophysiological pathways in (pre)diabetes may have differential effects on RA, RV and LA and LV structure and function [5]. Future research which improves the understanding of these differential effects, can therefore contribute to the development of RV targeted therapy.

### Confouding

Despite extensive adjustment for potential confounders, residual confounding cannot be fully ruled out. For example subclinical respiratory disease may be important in the association of (pre)diabetes and RA and RV structure and function [39]. Moreover, the use of body surface area to index the RA and LV mass may lead to overcorrection in the more obese (e.g. T2DM) population (with a consequential underestimation of the observed associations) [40].

### Limitations

The limitations of the present study may be its cross-sectional design which does not allow strong causal inferences. Reverse causality cannot be excluded, although, from a pathophysiological point of view it appears likely that (pre)diabetes can cause structural RA and RV changes and impaired RV function but not vice versa. Second, echocardiographic assessment of RA and RV structure and function could be hampered by several aspects; the crescent shape and anatomical position of the RV and RA adds complexity to measurement of structure and function (e.g. leading to increased random error of measurement); assessment of RA and RV pressures is less accurate than invasive testing and the use of a multiparameter approach to indicate RV diastolic function whereas a clear definition on RV diastolic function is absent [41, 42]. Other imaging modalities which are less hampered by anatomy (e.g. 3D echocardiography, cardiac MRI) or may be more sensitive to preclinical changes (e.g. deformation echocardiography using strain imaging or speckle tracking) or invasive testing were not available in this study. Third, the generalizability of our findings to other populations can be questioned; the study population primarily consisted of European Caucasians and within this cohort individuals with T2DM were well controlled for their diabetes (i.e. mean HbA1c 6.9%) and comorbid cardiovascular risk factors (i.e. use of antihypertensive and lipid-modifying medication in 68.8% and 74.1% respectively). Fourth, RV function was measured with use of TDI in the whole study population whereas only in a subset tricuspid inflow velocities and TAPSE were measured. Nevertheless, TDI measurements have been shown to be a reliable proxy measurement for RV function [35]. Fifth, clinical outcomes (e.g. incident heart failure, or cardiovascular death) were not available. However previous studies, in a general population, showed that RV structure and function were independent risk factors for incident heart failure and cardiovascular death [6, 43].

## Conclusions

In conclusion, in this population-based study (pre)diabetes is associated with structural RA and RV changes, and impaired RV systolic and diastolic function, independent of traditional cardiovascular risk factors. These associations were largely not statistically mediated by indices of LV structure, LV function or pulmonary pressure. This suggests that (pre)diabetes affects the RA and RV structure and function due to direct myocardial involvement. Although the absolute differences are small, this should increase the awareness that in patients with (pre)diabetes preclinical structural and functional changes already have taken place. The accumulated changes may alter the course of overt cardiac disease [44]. Although RV-targeted therapy is not available at the moment, these results suggest that future research should focus on the pathophysiological pathways of RA and RV impairment in (pre)diabetes.

## Supplementary information

**Additional file 1.** Supplemental methods.

**Additional file 2.** Supplemental tables.

## Data Availability

The datasets used and/or analyzed during the current study are available from the corresponding author on reasonable request.

## Abbreviations

LV: Left ventricular
LVMI: Left ventricular mass index
LVEF: Left ventricular ejection fraction
NGM: Normal glucose metabolism
RA: Right atrial
RV: Right ventricular
T2DM: Type 2 diabetes mellitus
TAPSE: Tricuspid annular plane systolic excursion
TDI: Tissue doppler imaging
TRmax grad: Maximum tricuspid gradient
