# Supplementary material for: Associations of (pre)diabetes with right ventricular and atrial structure and function: the Maastricht Study
Source: Cardiovasc Diabetol. 2020 Jun 15;19:88. doi: 10.1186/s12933-020-01055-y (PMC7296751; doi:10.1186/s12933-020-01055-y)
Supplement: Supplementary file 1 — Additional file 1. Supplemental methods. [file 12933_2020_1055_MOESM1_ESM.docx]

**Additional Methods**

**Associations of (pre)diabetes with right ventricular and atrial structure and function -The Maastricht Study-**

Pauline B.C. Linssen MD, Marja G.J. Veugen MD, PhD, Ronald M.A. Henry MD, PhD, Carla J.H. van der Kallen PhD, Abraham A. Kroon MD, PhD, Miranda T. Schram PhD, Hans-Peter Brunner-La Rocca MD, Coen D.A. Stehouwer MD, PhD

**Glucose metabolism status**

To determine glucose metabolism status, all participants (except those who used insulin) underwent a standardized 2-h 75g oral glucose tolerance test after an overnight fast. For safety reasons, participants with a fasting glucose level above 11.0 mmol/L, as determined by a finger prick, did not undergo the oral glucose tolerance test. For these individuals, fasting glucose level and information about diabetes medication were used to determine glucose metabolism status. Glucose metabolism status was defined according to the WHO 2006 criteria into normal glucose metabolism (NGM), impaired fasting glucose, impaired glucose tolerance (combined as prediabetes), and T2DM.[[1](#_ENREF_1)]

**Covariates**

Office blood pressure (Omron 705IT, Omron, Japan) and ambulatory 24 hour blood pressure (WatchBP O3, Microlife AG, Switzerland) were measured as described elsewhere.[[2](#_ENREF_2)] Fasting serum concentrations of total cholesterol, high density lipoprotein (HDL) cholesterol, triglycerides and creatinine were measured (Beckman Synchron LX20, Beckman Coulter inc., Brea USA).[[2](#_ENREF_2)] Cystatin C was measured by a particle enhanced immunoturbidimetric assay (Roche Cobas 8000, Roche diagnostics, Basel, Switzerland). Estimated glomerular filtration rate (eGFR) was estimated according to the Chronic Kidney Disease Epidemiology Collaboration equation based on both serum creatinine and serum cystatin C.[[3](#_ENREF_3)] Albuminuria, defined as an urinary albumin excretion ≥30 mg/24h, was determined (twice) as described elsewhere.[[4](#_ENREF_4)] Antihypertensive, lipid-modifying, and glucose-lowering medication use were assessed with a medication interview.[[2](#_ENREF_2)] Renin-angiotensin system modifying agents were defined angiotensin converting enzyme inhibitors, angiotensin II inhibitors and (or) renin inhibitors use. Waist circumference was measured midway between the lower rib margin and the iliac crest end-expiratory. Alcohol consumption, smoking status (never, former, current), prevalent cardiovascular disease and physical activity were determined by questionnaire.[[2](#_ENREF_2)] Alcohol consumption was categorized into non-consumers, low-consumers (≤7 and ≤14 glasses per week for females and males respectively) and high-consumers (>7 and >14 glasses per week for females and males respectively). Total and moderate to vigorous physical activity was assessed by a modified version of the Community Healthy Activities Model Program for Seniors (CHAMPS) questionnaire.[[5](#_ENREF_5)] Prevalent cardiovascular disease was defined as a self-reported history of myocardial infarction, or cerebrovascular infarction or hemorrhage, or percutaneous artery angioplasty of, or vascular surgery of the coronary, abdominal, peripheral or carotid arteries. Other clinical characteristics (i.e. body mass index, waist-to-hip ratio, presence of hypertension, and HbA1c) were obtained from physical examination and laboratory assessment as described elsewhere.[[2](#_ENREF_2)]

**Echocardiography**

Echocardiograms were obtained by research technicians according to a standardized protocol consisting of 2D, M-mode, color flow Doppler, pulsed and continuous wave Doppler, and Tissue Doppler recordings with use of echo equipment (Vivid E9 with 2.5-3.5 MHz and 4V transducer, GE Vingmed). All recordings were digitally stored and analyzed off-line (EchoPAC PC, version 112) by four researchers masked to glucose tolerance status.

End-systolic left atrial volume (LAV) was estimated in the four- and two-chamber view with use of the modified Simpson’s method.[[6](#_ENREF_6)] Preferably biplane, but otherwise uniplane, measurements were obtained (biplane was available in 97.6% of the participants). End-systolic right atrial volume (RAV) was estimated in the four-chamber view with use of planimetry.[[6](#_ENREF_6), [7](#_ENREF_7)] LAV and RAV were indexed to body surface area (BSA) calculated according to Mosteller.[[6](#_ENREF_6)]

End-diastolic and end-systolic interventricular septum (IVSD, IVSS), posterior wall thickness (PWTD, PWTS), and left ventricular (LV) diameters (LVEDD, LVESD) were determined in the parasternal long axis view, between the tip of the mitral leaflets and the chordae level perpendicular to the LV long axis.[[6](#_ENREF_6)] LV mass (LVM) was then calculated as 0.8*1.04*((LVEDD+IVSD+LVPWD)^3^–LVEDD^3^)+0.6. LVM was indexed (LVMI) by height^2,7^.[[6](#_ENREF_6), [8](#_ENREF_8), [9](#_ENREF_9)] Relative wall thickness (RWT) was calculated as ((2*PWTD)/LVEDD).[[6](#_ENREF_6)]

End-diastolic and end-systolic LV volumes (LVEDV, LVESV) were determined in the apical four- and two-chamber view with use of the modified Simpson’s method.[[6](#_ENREF_6)] Preferably biplane, but otherwise uniplane, measurements were obtained (biplane was available in 97.5% of the participants). LVEDV and LVESV were indexed to BSA. Systolic function was defined with the use of Simpson’s LV ejection fraction calculated from LVEDV and LVESV.[[6](#_ENREF_6)] The presence of wall motion abnormalities was evaluated by a trained researcher and checked by a senior cardiologist.

End-diastolic right ventricular (RV) diameter was measured in the four-chamber view optimized for the right ventricle, as the maximal short-axis dimension in the basal one third of the RV just above the closed tricuspid valve.[[6](#_ENREF_6), [7](#_ENREF_7)] In this view, the RV length was also measured from the plane of the tricuspid annulus to the RV apex.[[7](#_ENREF_7)]

Valve function was investigated in a qualitative and semi-quantitative way. The global severity of valve stenosis and regurgitation was based on valve morphology, color Doppler images, transvalvular (mean) gradient and jet velocity with the criteria specified in guidelines.[[10-15](#_ENREF_10)] Significant valvular dysfunction was defined as any moderate or severe valve stenosis or regurgitation of the aortic, mitral, tricuspid or pulmonary valve.

The LV outflow tract velocity was measured, with use of pulsed-wave Doppler in the apical three- or five-chamber view.[[10](#_ENREF_10), [11](#_ENREF_11)] The antegrade systolic velocity across the aortic valve was measured with use of continuous-wave Doppler in the apical three- or five-chamber view.[[10](#_ENREF_10), [11](#_ENREF_11)] In both pulsed and continuous waved signals, the maximal velocity, mean velocity, and velocity time integral were measured to calculate the maximum and mean gradients with use of the Bernoulli equation (i.e. 4v^2^).[[10](#_ENREF_10), [11](#_ENREF_11)]

Mitral inflow velocities were obtained with pulsed-wave Doppler in the apical four-chamber view with placement of the sample volume at the tips of the mitral leaflets.[[16](#_ENREF_16)] The peak flow velocity of the passive filling wave (E-wave) and active filling wave (A-wave) were measured and the E/A ratio was calculated.[[16](#_ENREF_16)] Furthermore, the deceleration time (DT) of the E-wave, and A-wave duration were measured.[[16](#_ENREF_16)]

Pulmonary venous inflow velocities were obtained with pulsed-wave Doppler in the apical four-chamber view with placement of the sample volume into the right upper pulmonary vein.^[^[^16^](#_ENREF_16)^]^ Peak systolic (S), anterograde diastolic (D), and atrial reversal (Ar) velocities were measured, as well as the duration of the Ar-wave.[[16](#_ENREF_16)] The difference between reversed A-wave duration and mitral A-wave duration was calculated.

Pulsed waved Doppler was obtained in the apical five-chamber view at the level of the LV outflow tract and the mitral inflow for assessment of closure to opening time (CTOT), isovolumetric contraction time (IVCT), ejection time (ET) and isovolumetric relaxation time (IVRT).[[16](#_ENREF_16)] The LV Tei index was calculated as (IVCT+IVRT)/ET.

Continuous wave Doppler recordings of the tricuspid flow were obtained in an apical four-chamber view.^[^[^7^](#_ENREF_7)^,^ [^16^](#_ENREF_16)^]^ Maximal tricuspid valve regurgitation velocity was measured and the maximal gradient was calculated with use of the Bernoulli equation.

Pulmonary valve regurgitation velocities at early diastole and end-diastole were obtained by use of continuous-wave Doppler in the parasternal short-axis view, with the placement of the cursor parallel in the right ventricular outflow tract.[[7](#_ENREF_7)] Until July 2012 these images were only obtained if there were signs of a significant pulmonary regurgitation during echocardiography. From July 2012 onwards, these were by default based on an extended protocol.

Pulsed Doppler tissue echocardiography was performed in the apical four-chamber view, with placement of the sample volume at the LV lateral and septal segment of the mitral annulus and at RV lateral segment of the tricuspid annulus. At each site the peak myocardial systolic (S’), early (E’) and late diastolic (A’) longitudinal velocities were measured.[[7](#_ENREF_7), [16](#_ENREF_16)] Furthermore, RV closure to opening time (CTOT) and ejection time (ET) were measured.[[6](#_ENREF_6), [7](#_ENREF_7)]The RV myocardial performance index, was calculated as (CTOT-ET)/ET.[[6](#_ENREF_6), [7](#_ENREF_7)] The mitral E/E’ ratio’s (septal, lateral and averaged) were calculated.

The presence of LV diastolic dysfunction was classified according to current guidelines (i.e. average E/e’>14, septal E’<7 or lateral E’<10, tricuspid regurgitation>2.8, LAVI>34).[[16](#_ENREF_16)] If ≥2 criteria were missing, diastolic function was classified as not specified.

In July 2012 the standardized echocardiography recording protocol was extended, with addition of vena cava inferior, short axis RV outflow tract, and TAPSE recordings.

The inferior vena cava (IVC) diameter was measured in subcostal M-mode view at end-expiration, just proximal to the junction of the hepatic veins, near the ostium of the right atrium. The IVC collapse during sniff or inspiration is defined as the relative change of the IVC diameter.[[7](#_ENREF_7)]

Tricuspid inflow velocities were obtained with pulsed wave Doppler in the apical four-chamber view with placement of the sample volume at the tips of the tricuspids leaflets.[[7](#_ENREF_7)] The peak flow velocity of the passive filling wave (E-wave) and active filling wave (A-wave) were measured and the tricuspid E/A ratio was calculated.[[7](#_ENREF_7)]

The tricuspid annular plane systolic excursion (TAPSE), was obtained in the apical four-chamber M-mode view at the lateral tricuspid annulus. The longitudinal motion at peak systole was measured.[[6](#_ENREF_6), [7](#_ENREF_7)]

Reproducibility of the analysis was assessed in 12 individuals (50% women; 57.8±11.5 years; four T2DM, four pre-diabetes) who were analysed by four observers. Intraclass correlation coefficients of observed agreement are described as below.

| Variable | ICC (95% CI) | Variable | ICC (95% CI) | Variable | ICC (95% CI) |
| --- | --- | --- | --- | --- | --- |
| LVEDD | 0.88 (0.75-0.96) | E peak mitral | 0.95 (0.86-0.98) | RVET | 0.88 (0.73-0.97) |
| LVESD | 0.94 (0.85-0.98) | A peak mitral | 0.83 (0.53-0.95) | S’ RV | 0.79 (0.35-0.95) |
| IVSD | 0.61 (0.29-0.85) | Dec. time E peak mitral | 0.86 (0.71-0.95) | E’ RV | 0.62 (0.14-0.89) |
| PWTD | 0.71 (0.45-0.89) | A peak duration mitral | 0.92 (0.82-0.98) | A’ RV | 0.89 (0.48-0.98) |
| LVEDV | 0.59 (0.16-0.85) | S peak | 0.86 (0.53-0.96) | S’ LV septal | 0.87 (0.48-0.97) |
| LVESV | 0.66 (0.23-0.88) | D peak | 0.88 (0.62-0.96) | E’ LV septal | 0.96 (0.81-0.99) |
| LAV | 0.83 (0.59-0.94) | A peak reverse velocity | 0.70 (0.44-0.89) | A’ LV septal | 0.87 (0.56-0.96) |
| RAV | 0.81 (0.52-0.94) | A reverse duration | 0.70 (0.43-0.89) | S’ LV lateral | 0.86 (0.48-0.96) |
| RV diameter | 0.93 (0.85-0.98) | Tricuspid regurgitation | 0.70 (0.44-0.88) | E’ LV lateral | 0.93 (0.66-0.98) |
| RV length | 0.83 (0.62-0.95) | CTOT tricuspid | 0.96 (0.91-0.99) | A’ LV lateral | 0.61 (0.14-0.87) |

The data are given in intraclass correlation coefficients (ICC) with their 95% confidence interval (95% CI).

**References**

1. World Health Organization: **Definition and diagnosis of diabetes mellitus and intermediate hyperglycemia**. In*.*; 2006.

2. Schram MT, Sep SJ, van der Kallen CJ, Dagnelie PC, Koster A, Schaper N, Henry RM, Stehouwer CD: **The Maastricht Study: an extensive phenotyping study on determinants of type 2 diabetes, its complications and its comorbidities**. *European journal of epidemiology* 2014, **29**(6):439-451.

3. Inker LA, Schmid CH, Tighiouart H, Eckfeldt JH, Feldman HI, Greene T, Kusek JW, Manzi J, Van Lente F, Zhang YL *et al*: **Estimating glomerular filtration rate from serum creatinine and cystatin C**. *The New England journal of medicine* 2012, **367**(1):20-29.

4. Martens RJ, Kimenai DM, Kooman JP, Stehouwer CD, Tan FE, Bekers O, Dagnelie PC, van der Kallen CJ, Kroon AA, Leunissen KM *et al*: **Estimated Glomerular Filtration Rate and Albuminuria Are Associated with Biomarkers of Cardiac Injury in a Population-Based Cohort Study: The Maastricht Study**. *Clinical chemistry* 2017, **63**(4):887-897.

5. Stewart AL, Mills KM, King AC, Haskell WL, Gillis D, Ritter PL: **CHAMPS physical activity questionnaire for older adults: outcomes for interventions**. *Medicine and science in sports and exercise* 2001, **33**(7):1126-1141.

6. Lang RM, Badano LP, Mor-Avi V, Afilalo J, Armstrong A, Ernande L, Flachskampf FA, Foster E, Goldstein SA, Kuznetsova T *et al*: **Recommendations for cardiac chamber quantification by echocardiography in adults: an update from the American Society of Echocardiography and the European Association of Cardiovascular Imaging**. *Journal of the American Society of Echocardiography : official publication of the American Society of Echocardiography* 2015, **28**(1):1-39 e14.

7. Rudski LG, Lai WW, Afilalo J, Hua L, Handschumacher MD, Chandrasekaran K, Solomon SD, Louie EK, Schiller NB: **Guidelines for the echocardiographic assessment of the right heart in adults: a report from the American Society of Echocardiography endorsed by the European Association of Echocardiography, a registered branch of the European Society of Cardiology, and the Canadian Society of Echocardiography**. *Journal of the American Society of Echocardiography : official publication of the American Society of Echocardiography* 2010, **23**(7):685-713; quiz 786-688.

8. de Simone G, Daniels SR, Devereux RB, Meyer RA, Roman MJ, de Divitiis O, Alderman MH: **Left ventricular mass and body size in normotensive children and adults: assessment of allometric relations and impact of overweight**. *Journal of the American College of Cardiology* 1992, **20**(5):1251-1260.

9. Cuspidi C, Meani S, Negri F, Giudici V, Valerio C, Sala C, Zanchetti A, Mancia G: **Indexation of left ventricular mass to body surface area and height to allometric power of 2.7: is the difference limited to obese hypertensives?** *Journal of human hypertension* 2009, **23**(11):728-734.

10. Baumgartner H, Hung J, Bermejo J, Chambers JB, Edvardsen T, Goldstein S, Lancellotti P, LeFevre M, Miller F, Jr., Otto CM: **Recommendations on the Echocardiographic Assessment of Aortic Valve Stenosis: A Focused Update from the European Association of Cardiovascular Imaging and the American Society of Echocardiography**. *Journal of the American Society of Echocardiography : official publication of the American Society of Echocardiography* 2017, **30**(4):372-392.

11. Baumgartner H, Hung J, Bermejo J, Chambers JB, Evangelista A, Griffin BP, Iung B, Otto CM, Pellikka PA, Quinones M *et al*: **Echocardiographic assessment of valve stenosis: EAE/ASE recommendations for clinical practice**. *Journal of the American Society of Echocardiography : official publication of the American Society of Echocardiography* 2009, **22**(1):1-23; quiz 101-102.

12. Lancellotti P, Moura L, Pierard LA, Agricola E, Popescu BA, Tribouilloy C, Hagendorff A, Monin JL, Badano L, Zamorano JL *et al*: **European Association of Echocardiography recommendations for the assessment of valvular regurgitation. Part 2: mitral and tricuspid regurgitation (native valve disease)**. *European journal of echocardiography : the journal of the Working Group on Echocardiography of the European Society of Cardiology* 2010, **11**(4):307-332.

13. Lancellotti P, Tribouilloy C, Hagendorff A, Moura L, Popescu BA, Agricola E, Monin JL, Pierard LA, Badano L, Zamorano JL *et al*: **European Association of Echocardiography recommendations for the assessment of valvular regurgitation. Part 1: aortic and pulmonary regurgitation (native valve disease)**. *European journal of echocardiography : the journal of the Working Group on Echocardiography of the European Society of Cardiology* 2010, **11**(3):223-244.

14. Lancellotti P, Tribouilloy C, Hagendorff A, Popescu BA, Edvardsen T, Pierard LA, Badano L, Zamorano JL, Scientific Document Committee of the European Association of Cardiovascular I: **Recommendations for the echocardiographic assessment of native valvular regurgitation: an executive summary from the European Association of Cardiovascular Imaging**. *European heart journal cardiovascular Imaging* 2013, **14**(7):611-644.

15. Zoghbi WA, Adams D, Bonow RO, Enriquez-Sarano M, Foster E, Grayburn PA, Hahn RT, Han Y, Hung J, Lang RM *et al*: **Recommendations for Noninvasive Evaluation of Native Valvular Regurgitation: A Report from the American Society of Echocardiography Developed in Collaboration with the Society for Cardiovascular Magnetic Resonance**. *Journal of the American Society of Echocardiography : official publication of the American Society of Echocardiography* 2017, **30**(4):303-371.

16. Nagueh SF, Smiseth OA, Appleton CP, Byrd BF, 3rd, Dokainish H, Edvardsen T, Flachskampf FA, Gillebert TC, Klein AL, Lancellotti P *et al*: **Recommendations for the Evaluation of Left Ventricular Diastolic Function by Echocardiography: An Update from the American Society of Echocardiography and the European Association of Cardiovascular Imaging**. *Journal of the American Society of Echocardiography : official publication of the American Society of Echocardiography* 2016, **29**(4):277-314.
