# Supplementary material for: Associations of (pre)diabetes with right ventricular and atrial structure and function: the Maastricht Study
Source: Cardiovasc Diabetol. 2020 Jun 15;19:88. doi: 10.1186/s12933-020-01055-y (PMC7296751; doi:10.1186/s12933-020-01055-y)
Supplement: Supplementary file 2 — Additional file 2. Supplemental tables. [file 12933_2020_1055_MOESM2_ESM.docx]

**Additional Tables**

**Associations of (pre)diabetes with right ventricular and atrial structure and function -The Maastricht Study-**

Pauline B.C. Linssen MD, Marja G.J. Veugen MD, PhD, Ronald M.A. Henry MD, PhD, Carla J.H. van der Kallen PhD, Abraham A. Kroon MD, PhD, Miranda T. Schram PhD, Hans-Peter Brunner-La Rocca MD, Coen D.A. Stehouwer MD, PhD

| **Supplemental table 1a: Clinical characteristics of the study population with two-dimensional echocardiography and individuals excluded from analyses** | | | | | | | | | | | | | | | | | | | | |
| --- | --- | --- | --- | --- | --- | --- | --- | --- | --- | --- | --- | --- | --- | --- | --- | --- | --- | --- | --- | --- |
| **Variable** | **Normal glucose metabolism** | | | | | **Prediabetes** | | | | | **Type 2 Diabetes** | | | | | **Total study population** | | | | |
|  | **Included (n=426)** | | **Excluded (n=132)** | | **P** | **Included (n=142)** | | **Excluded (n=46)** | | **P** | **Included (n=224)** | | **Excluded (n=107)** | | **P** | **Included (n=792)** | | **Excluded (n=285)** | | **P** |
| Age, years | 57.3 | ±8.0 | 57.1 | ±9.3 | 0.83 | 62.5 | ±7.5 | 60.5 | ±7.6 | 0.13 | 62.6 | ±7.6 | 64.5 | ±6.5 | 0.02 | 59.7 | ±8.3 | 60.4 | ±8.7 | 0.21 |
| Women,% | 233 | (54.7) | 79 | (59.8) | 0.30 | 57 | (40.1) | 19 | (41.3) | 0.89 | 71 | (31.7) | 33 | (30.8) | 0.88 | 361 | (45.6) | 131 | (46.0) | 0.91 |
| BMI, kg/m2 | 25.5 | [23.2-27.8] | 25.1 | [23.0-27.5] | 0.69 | 27.4 | [25.6-29.8] | 26.8 | [23.8-29.0] | 0.11 | 29.3 | [26.2-32.2] | 29.4 | [26.8-32.9] | 0.22 | 26.8 | [24.3-29.5] | 26.8 | [24.2-30.1] | 0.34 |
| Waist circumference, cm |  |  |  |  |  |  |  |  |  |  |  |  |  |  |  |  |  |  |  |  |
| Men | 96 | [91-103] | 97 | [89-104] | 0.50 | 102 | [96-110] | 97 | [90-105] | 0.15 | 106 | [99-113] | 109 | [101-119] | 0.03 | 101 | [94-108] | 103 | [95-111] | 0.12 |
| Women | 87 | [79-94] | 86 | [79-93] | 0.72 | 94 | [85-100] | 89 | [81-99] | 0.24 | 101 | [91-113] | 103 | [90-113] | 0.72 | 90 | [82-99] | 89 | [81-97] | 0.84 |
| Waist-to hip ratio | 0.91 | ±0.08 | 0.90 | ±0.09 | 0.32 | 0.97 | ±0.08 | 0.95 | ±0.08 | 0.32 | 1.01 | ±0.08 | 1.01 | ±0.09 | 0.54 | 0.95 | ±0.09 | 0.95 | ±0.10 | 0.54 |
| Office systolic blood pressure, mmHg | 131.1 | ±16.5 | 133.2 | ±18.8 | 0.21 | 140.0 | ±17.2 | 136.2 | ±15.7 | 0.19 | 145.0 | ±18.3 | 146.6 | ±18.9 | 0.48 | 136.6 | ±18.2 | 138.7 | ±19.3 | 0.10 |
| Office diastolic blood pressure mmHg | 75.2 | ±9.7 | 75.8 | ±10.4 | 0.49 | 79.3 | ±10.3 | 77.4 | ±9.6 | 0.27 | 78.0 | ±9.3 | 78.0 | ±10.6 | 0.95 | 76.7 | ±9.8 | 76.9 | ±10.4 | 0.76 |
| 24h average ambulatory systolic  blood pressure, mmHg | 116.3 | ±10.6 | 118.4 | ±11.4 | 0.06 | 121.6 | ±12.8 | 121.4 | ±14.5 | 0.92 | 122.1 | ±11.5 | 124.4 | ±14.8 | 0.15 | 118.9 | ±11.6 | 121.1 | ±13.5 | 0.01 |
| 24h average ambulatory diastolic  blood pressure, mmHg | 73.6 | ±7.0 | 75.1 | ±7.3 | 0.05 | 74.8 | ±8.0 | 76.1 | ±7.6 | 0.35 | 73.4 | ±7.1 | 73.3 | ±7.5 | 0.91 | 73.8 | ±7.2 | 74.6 | ±7.5 | 0.12 |
| Glucose metabolism status | - | - | - | - | - | - | - | - | - | - | - | - | - | - | - | 426/142/224  (53.8/17.9/28.3) | | 132/46/107  (46.3/16.1/37.5) | | 0.01 |
| Hypertension, % | 169 | (39.7) | 54 | (40.9) | 0.80 | 98 | (69.0) | 24 | (53.3) | 0.05 | 187 | (83.5) | 100 | (93.5) | 0.01 | 454 | (57.3) | 178 | (62.7) | 0.12 |
| Fasting glucose, mmol/L | 5.2 | ±0.4 | 5.2 | ±0.4 | 0.19 | 6.0 | ±0.5 | 6.0 | ±0.6 | 0.65 | 7.9 | ±2.3 | 7.9 | ±1.7 | 0.97 | 6.1 | 1.7 | 6.4 | ±1.7 | 0.03 |
| 2h postload glucose | 5.3 | ±1.2 | 5.5 | ±1.1 | 0.04 | 8.2 | ±1.8 | 7.8 | ±1.7 | 0.20 | 14.0 | ±3.4 | 14.7 | ±4.1 | 0.15 | 7.9 | 4.1 | 8.6 | ±4.7 | 0.02 |
| HbA1c, mmol/mol | 37.1 | ±3.5 | 37.4 | ±3.8 | 0.54 | 40.4 | ±4.2 | 39.1 | ±5.2 | 0.08 | 51.7 | ±11.2 | 53.3 | ±11.0 | 0.22 | 41.9 | 9.2 | 43.7 | ±10.6 | 0.01 |
| HbA1c, % | 5.5 | ±0.3 | 5.6 | ±0.3 | 0.44 | 5.8 | ±0.4 | 5.7 | ±0.5 | 0.10 | 6.9 | ±1.0 | 7.0 | ±1.0 | 0.23 | 6.0 | 0.8 | 6.1 | ±1.0 | 0.01 |
| Total cholesterol, mmol/L | 5.6 | ±1.0 | 5.5 | ±1.1 | 0.30 | 5.5 | ±1.2 | 5.4 | ±1.2 | 0.60 | 4.5 | ±1.0 | 4.3 | ±1.1 | 0.21 | 5.3 | ±1.1 | 5.0 | ±1.2 | 0.01 |
| HDL cholesterol, mmol/l |  |  |  |  |  |  |  |  |  |  |  |  |  |  |  |  |  |  |  |  |
| Men | 1.3 | ±0.3 | 1.3 | ±0.4 | 0.63 | 1.2 | ±0.3 | 1.2 | ±0.3 | 0.75 | 1.1 | ±0.3 | 1.0 | ±0.2 | 0.01 | 1.2 | ±0.3 | 1.1 | ±0.3 | 0.03 |
| Women | 1.7 | ±0.5 | 1.6 | ±0.4 | 0.06 | 1.6 | ±0.4 | 1.5 | ±0.3 | 0.45 | 1.4 | ±0.4 | 1.3 | ±0.4 | 0.36 | 1.6 | ±0.5 | 1.5 | ±0.4 | 0.01 |
| Total to HDL cholesterol ratio | 4.0 | ±1.3 | 4.0 | ±1.2 | 0.79 | 4.3 | ±1.3 | 4.2 | ±1.2 | 0.94 | 4.0 | ±1.1 | 4.2 | ±1.2 | 0.11 | 4.1 | ±1.3 | 4.1 | ±1.2 | 0.52 |
| LDL cholesterol, mmol/l | 3.6 | ±0.9 | 3.5 | ±1.0 | 0.86 | 3.4 | ±1.0 | 3.4 | ±1.1 | 0.83 | 2.5 | ±0.9 | 2.5 | ±0.9 | 0.46 | 3.2 | ±1.0 | 3.1 | ±1.1 | 0.06 |
| Triglycerides, mmol/l | 1.0 | [0.8-1.5] | 1.0 | [0.8-1.4] | 0.20 | 1.4 | [0.9-1.9] | 1.3 | [1.0-1.9] | 0.66 | 1.7 | [1.2-2.3] | 1.6 | [1.2-2.3] | 0.91 | 1.2 | [0.9-1.8] | 1.3 | [0.9-1.8] | 0.89 |
| eGFR, ml/min/1.73 m^2^ | 90.8 | ±13.8 | 91.2 | ±13.8 | 0.76 | 85.0 | ±14.4 | 87 | ±13 | 0.41 | 85.2 | ±17.2 | 82.0 | ±18.3 | 0.13 | 88.2 | ±15.2 | 87.0 | ±16.1 | 0.30 |
| albuminuria, % | 15 | (3.5) | 5 | (3.9) | 0.83 | 10 | (7.0) | 3 | (6.7) | 0.93 | 38 | (17.0) | 23 | (23.0) | 0.20 | 63 | (8.0) | 31 | (11.4) | 0.08 |
| Smoking (never, former, current), % | 153/198/75  (35.9/46.5/17.6) | | 46/59/22  (36.2/46.5/17.3) | | 1.00 | 41/85/16  (28.9/59.9/11.3) | | 13/23/7  (30.2/53.5/16.3) | | 0.63 | 59/128/37  (26.3/57.1/16.5) | | 16/64/12  (17.4/69.6/13.0) | | 0.11 | 253/411/128  (31.9/51.9/16.2) | | 75/146/41  (28.6/55.7/15.6) | | 0.53 |
| Alcohol consumption (none, low, high), % | 52/226/145  (12.3/53.4/34.3) | | 22/67/38  (17.3/52.8/29.9) | | 0.30 | 21/70/51  (14.8/49.3/35.9) | | 3/31/9  (7.0/72.1/20.9) | | 0.03 | 66/109/48  (29.6/48.9/21.5) | | 30/45/19  (31.9/47.9/20.2) | | 0.91 | 139/405/244  (17.6/51.4/31.0) | | 55/143/66  (20.8/54.2/25.0) | | 0.15 |
| Moderate to vigorous physical activity, h/wk | 5.3 | [3.0-8.5] | 5.9 | [3.8-9.0] | 0.40 | 4.5 | [2.0-6.8] | 3.4 | [1.5-7.0] | 0.70 | 3.8 | [1.6-6.5] | 3.0 | [1.5-5.9] | 0.20 | 4.5 | [2.3-7.8] | 4.5 | [2.1-7.8] | 0.52 |
| Prior cardiovascular disease, % | 44 | (10.3) | 14 | (12.1) | 0.59 | 23 | (16.2) | 8 | (20.0) | 0.57 | 56 | (25.0) | 34 | (40.5) | 0.01 | 123 | (15.5) | 56 | (23.3) | 0.01 |
| Antihypertensive medication use, % | 97 | (22.8) | 24 | (18.2) | 0.26 | 68 | (47.9) | 17 | (37.0) | 0.20 | 154 | (68.8) | 86 | (80.4) | 0.03 | 319 | (40.3) | 127 | (44.6) | 0.21 |
| Ras inhibitors, % | 64 | (15.0) | 18 | (13.6) | 0.69 | 49 | (34.5) | 10 | (21.7) | 0.11 | 124 | (55.4) | 73 | (68.2) | 0.03 | 237 | (29.9) | 101 | (35.4) | 0.09 |
| Beta- blockers, % | 36 | (8.5) | 10 | (7.6) | 0.75 | 32 | (22.5) | 10 | (21.7) | 0.91 | 81 | (36.2) | 34 | (31.8) | 0.43 | 149 | (18.8) | 54 | (18.9) | 0.96 |
| Diuretics, % | 29 | (6.8) | 10 | (7.6) | 0.76 | 29 | (20.4) | 5 | (10.9) | 0.14 | 60 | (26.8) | 31 | (29.0) | 0.68 | 118 | (14.9) | 46 | (16.1) | 0.62 |
| Calcium antagonists, % | 15 | (3.5) | 2 | (1.5) | 0.24 | 11 | (7.7) | 5 | (10.9) | 0.51 | 35 | (15.6) | 30 | (28.0) | 0.01 | 61 | (7.7) | 37 | (13.0) | 0.01 |
| Diabetes medication use, % | - | - | - | - | - | - | - | - | - | - | 166 | (74.1) | 93 | (86.9) | 0.01 | - | - | - | - | - |
| Insulin, % | - | - | - | - | - | - | - | - | - | - | 40 | (17.9) | 27 | (25.2) | 0.12 | - | - | - | - | - |
| Metformin, % | - | - | - | - | - | - | - | - | - | - | 151 | (67.4) | 79 | (73.8) | 0.24 | - | - | - | - | - |
| Sulfonylureas, % | - | - | - | - | - | - | - | - | - | - | 41 | (18.3) | 29 | (27.1) | 0.07 | - | - | - | - | - |
| Thiazolidinediones, % | - | - | - | - | - | - | - | - | - | - | 2 | (0.9) | 4 | (3.7) | 0.07 | - | - | - | - | - |
| GLP-1 analogs, % | - | - | - | - | - | - | - | - | - | - | 2 | (0.9) | 2 | (1.9) | 0.45 | - | - | - | - | - |
| DPP-4 inhibitors, % | - | - | - | - | - | - | - | - | - | - | 5 | (2.2) | 3 | (2.8) | 0.75 | - | - | - | - | - |
| Lipid modifying medication use, % | 68 | (16.0) | 23 | (17.4) | 0.69 | 54 | (38.0) | 13 | (28.3) | 0.23 | 166 | (74.1) | 83 | (77.6) | 0.50 | 288 | (36.4) | 119 | (41.8) | 0.11 |
| Data are presented as n (%), mean ± standard deviation or median [interquartile rage]. Abbreviations BMI, body mass index; HDL, high density lipoprotein; LDL, Low density lipoprotein; eGFR, estimated glomerular filtration rate;  Ras, renin-angiotensin system inhibitor; GLP-1, glucagon-like peptide 1; DPP-4, dipeptidyl peptidase-4. All tested with independent sample T-test, Mann Whitney U or Chi-square test as appropriate | | | | | | | | | | | | | | | | | | | | |

| **Supplemental table 1b: Clinical characteristics of the study population with TDI echocardiography and individuals excluded from analyses** | | | | | | | | | | | | | | | | | | | | |
| --- | --- | --- | --- | --- | --- | --- | --- | --- | --- | --- | --- | --- | --- | --- | --- | --- | --- | --- | --- | --- |
| **Variable** | **Normal glucose metabolism** | | | | | **Pre-diabetes** | | | | | **Type 2 Diabetes** | | | | | **Total study population** | | | | |
|  | **Included (n=416)** | | **Excluded (n=142)** | | **P** | **Included (n=134)** | | **Excluded (n=54)** | | **P** | **Included (n=198)** | | **Excluded (n=133)** | | **P** | **Included (n=748)** | | **Excluded (n=329)** | | **P** |
| Age, years | 57.1 | ±8.1 | 57.7 | ±9.1 | 0.48 | 62.1 | ±7.6 | 61.8 | ±7.6 | 0.82 | 62.8 | ±7.5 | 63.8 | ±6.9 | 0.21 | 59.5 | ±8.3 | 60.8 | ±8.5 | 0.02 |
| Women,% | 222 | (53.4) | 90 | (63.4) | 0.04 | 51 | (38.1) | 25 | (46.3) | 0.30 | 72 | (31.3) | 42 | (31.6) | 0.96 | 335 | (44.8) | 157 | (47.7) | 0.37 |
| BMI, kg/m2 | 25.3 | [23.2-27.5] | 25.6 | [23.1-28.7] | 0.06 | 27.1 | [25.4-29.4] | 27.7 | [24.5-31.9] | 0.32 | 28.7 | [25.9-31.6] | 30.1 | [27.1-33.9] | <0.01 | 26.4 | [24.0-29.1] | 27.7 | [24.8-31.7] | <0.01 |
| Waist circumference, cm |  |  |  |  |  |  |  |  |  |  |  |  |  |  |  |  |  |  |  |  |
| Men | 96 | [90-102] | 100 | [92-106] | 0.04 | 101 | [96-109] | 104 | [94-115] | 0.38 | 105 | [98-112] | 110 | [102-119] | <0.01 | 100 | [93-107] | 105 | [97-114] | <0.01 |
| Women | 86 | [79-92] | 86 | [79-94] | 0.22 | 93 | [84-99] | 96 | [82-102] | 0.46 | 101 | [89-107] | 105 | [91-118] | 0.08 | 88 | [81-97] | 92 | [82-104] | <0.01 |
| Waist-to hip ratio | 0.91 | ±0.08 | 0.91 | ±0.09 | 0.70 | 0.96 | ±0.08 | 0.97 | ±0.09 | 0.72 | 1.00 | ±0.08 | 1.02 | ±0.09 | 0.03 | 0.94 | ±0.09 | 0.96 | ±0.10 | <0.01 |
| Office systolic blood pressure, mmHg | 131.0 | ±16.4 | 133.3 | ±18.6 | 0.17 | 140.7 | ±17.0 | 135.0 | ±16.1 | 0.04 | 145.3 | ±18.6 | 145.8 | ±18.4 | 0.82 | 136.5 | ±18.3 | 138.6 | ±19.0 | 0.09 |
| Office diastolic blood pressure mmHg | 75.1 | ±9.8 | 75.9 | ±10.1 | 0.43 | 79.6 | ±10.0 | 77.0 | ±10.2 | 0.12 | 77.9 | ±9.7 | 78.1 | ±9.8 | 0.88 | 76.7 | ±10.0 | 77.0 | ±10.0 | 0.65 |
| 24h average ambulatory systolic  blood pressure, mmHg | 116.5 | ±10.7 | 117.7 | ±11.1 | 0.25 | 122.0 | ±12.6 | 120.5 | ±14.9 | 0.50 | 122.3 | ±11.8 | 123.7 | ±13.8 | 0.34 | 119.0 | ±11.7 | 120.5 | ±13.1 | 0.06 |
| 24h average ambulatory diastolic  blood pressure, mmHg | 73.5 | ±7.0 | 75.3 | ±7.3 | 0.02 | 75.3 | ±8.0 | 74.8 | ±7.7 | 0.73 | 73.1 | ±7.2 | 73.9 | ±7.2 | 0.35 | 73.7 | ±7.3 | 74.7 | ±7.3 | 0.07 |
| Glucose metabolism status | - | - | - | - | - | - | - | - | - | - | - | - | - | - | - | 416/134/198  (55.6/17.9/26.5) | | 142/54/133  (43.2/16.4/40.4) | | <0.01 |
| Hypertension, % | 161 | (38.7) | 62 | (43.7) | 0.30 | 86 | (64.2) | 36 | (67.9) | 0.63 | 162 | (81.8) | 125 | (94.0) | 0.01 | 409 | (54.7) | 223 | (68.0) | <0.01 |
| Fasting glucose, mmol/L | 5.2 | ±0.4 | 5.3 | ±0.4 | 0.05 | 6.0 | ±0.5 | 5.9 | ±0.6 | 0.41 | 7.7 | ±2.0 | 8.2 | ±2.3 | 0.06 | 6.0 | ±1.5 | 6.6 | ±2.0 | <0.01 |
| 2h postload glucose | 5.3 | ±1.1 | 5.7 | ±1.1 | <0.01 | 8.2 | ±1.8 | 7.9 | ±1.7 | 0.25 | 14.1 | ±3.5 | 14.5 | ±3.9 | 0.41 | 7.8 | ±4.1 | 8.8 | ±4.6 | <0.01 |
| HbA1c, mmol/mol | 37.0 | ±3.5 | 37.9 | ±3.7 | <0.01 | 40.2 | ±4.3 | 39.9 | ±4.8 | 0.68 | 50.9 | ±10.4 | 54.2 | ±12.0 | <0.01 | 41.2 | ±8.6 | 44.8 | ±11.3 | <0.01 |
| HbA1c, % | 5.5 | ±0.3 | 5.6 | ±0.3 | <0.01 | 5.8 | ±0.4 | 5.8 | ±0.4 | 0.70 | 6.8 | ±1.0 | 7.1 | ±1.1 | <0.01 | 5.9 | ±0.8 | 6.3 | ±1.0 | <0.01 |
| Total cholesterol, mmol/L | 5.6 | ±1.0 | 5.6 | ±1.1 | 0.81 | 5.5 | ±1.1 | 5.4 | ±1.4 | 0.44 | 4.5 | ±1.0 | 4.3 | ±1.1 | 0.17 | 5.3 | ±1.1 | 5.0 | ±1.3 | <0.01 |
| HDL cholesterol, mmol/l |  |  |  |  |  |  |  |  |  |  |  |  |  |  |  |  |  |  |  |  |
| Men | 1.3 | ±0.3 | 1.2 | ±0.3 | 0.07 | 1.3 | ±0.3 | 1.2 | ±0.3 | 0.46 | 1.1 | ±0.3 | 1.0 | ±0.2 | <0.01 | 1.2 | ±0.3 | 1.1 | ±0.3 | <0.01 |
| Women | 1.7 | ±0.5 | 1.5 | ±0.3 | <0.01 | 1.6 | ±0.4 | 1.4 | ±0.4 | 0.07 | 1.4 | ±0.4 | 1.3 | ±0.4 | 0.14 | 1.6 | ±0.5 | 1.4 | ±0.4 | <0.01 |
| Total to HDL cholesterol ratio | 4.0 | ±1.3 | 4.2 | ±1.2 | 0.20 | 4.2 | ±1.3 | 4.3 | ±1.3 | 0.72 | 4.0 | ±1.1 | 4.3 | ±1.1 | 0.03 | 4.0 | ±1.3 | 4.2 | ±1.2 | 0.02 |
| LDL cholesterol, mmol/l | 3.5 | ±0.9 | 3.6 | ±1.0 | 0.37 | 3.4 | ±1.0 | 3.4 | ±1.2 | 0.74 | 2.6 | ±0.9 | 2.5 | ±0.9 | 0.35 | 3.3 | ±1.0 | 3.1 | ±1.1 | 0.03 |
| Triglycerides, mmol/l | 1.0 | [0.7-1.4] | 1.0 | [0.8-1.5] | 0.58 | 1.4 | [1.1-1.9] | 1.4 | [0.9-1.9] | 0.70 | 1.6 | [1.1-2.1] | 1.7 | [1.2-2.4] | 0.34 | 1.2 | [0.9-1.8] | 1.3 | [0.9-1.9] | <0.01 |
| eGFR, ml/min/1.73 m^2^ | 91.2 | ±13.5 | 90.0 | ±14.9 | 0.40 | 85.9 | ±14.0 | 84.4 | ±14.3 | 0.51 | 85.5 | ±17.0 | 82.2 | ±18.4 | 0.10 | 88.7 | ±14.8 | 85.9 | ±16.6 | <0.01 |
| albuminuria, % | 16 | (3.8) | 4 | (2.9) | 0.61 | 9 | (6.7) | 4 | (7.5) | 0.84 | 34 | (17.2) | 27 | (21.4) | 0.34 | 59 | (7.9) | 35 | (11.1) | 0.09 |
| Smoking (never, former, current), % | 158/189/69  (38.0/45.4/16.6) | | 41/68/28  (29.9/49.6/20.4) | | 0.21 | 41/80/13  (30.6/59.7/9.7) | | 13/28/10  (25.5/54.9/19.6) | | 0.18 | 54/115/29  (27.3/58.1/14.6) | | 21/77/20  (17.8/65.3/16.9) | | 0.16 | 253/384/111  (33.8/51.3/14.8) | | 75/173/58  (24.5/56.5/19.0) | | <0.01 |
| Alcohol consumption (none, low, high), % | 54/227/132  (13.1/55.0/32.0) | | 20/66/51  (14.6/48.2/37.2) | | 0.38 | 20/66/48  (14.9/49.3/35.8) | | 4/35/12  (7.8/68.6/23.5) | | 0.06 | 56/97/44  (28.4/49.2/22.3) | | 40/57/23  (33.3/47.5/19.2) | | 0.61 | 130/390/224  (17.5/52.4/30.1) | | 64/158/86  (20.8/51.3/27.9) | | 0.43 |
| Moderate to vigorous physical activity, h/wk | 5.5 | [3.0-8.8] | 5.3 | [3.0-8.6] | 0.54 | 4.5 | [2.3-7.5] | 3.0 | [1.5-6.8] | 0.03 | 3.8 | [1.8-6.8] | 3.0 | [0.9-5.3] | 0.01 | 4.8 | [2.8-8.0] | 3.8 | [1.5-6.9] | <0.01 |
| Prior cardiovascular disease, % | 43 | (10.3) | 15 | (11.9) | 0.62 | 20 | (14.9) | 11 | (22.9) | 0.21 | 48 | (24.2) | 42 | (38.2) | 0.01 | 111 | (14.8) | 68 | (23.9) | 0.01 |
| Antihypertensive medication use, % | 89 | (21.4) | 32 | (22.5) | 0.78 | 56 | (41.8) | 29 | (53.7) | 0.14 | 131 | (66.2) | 109 | (82.0) | <0.01 | 276 | (36.9) | 170 | (51.7) | <0.01 |
| Ras inhibitors, % | 60 | (14.4) | 22 | (15.5) | 0.76 | 40 | (29.9) | 19 | (35.2) | 0.48 | 106 | (53.5) | 91 | (68.4) | <0.01 | 206 | (27.5) | 132 | (40.1) | <0.01 |
| Beta- blockers, % | 31 | (7.5) | 15 | (10.6) | 0.24 | 29 | (21.6) | 13 | (24.1) | 0.72 | 67 | (33.8) | 48 | (36.1) | 0.67 | 127 | (17.0) | 76 | (23.1) | 0.02 |
| Diuretics, % | 25 | (6.0) | 14 | (9.9) | 0.12 | 24 | (17.9) | 10 | (18.5) | 0.92 | 43 | (21.7) | 48 | (36.1) | <0.01 | 92 | (12.3) | 72 | (21.9) | <0.01 |
| Calcium antagonists, % | 14 | (3.4) | 3 | (2.1) | 0.45 | 8 | (6.0) | 8 | (14.8) | 0.05 | 26 | (13.1) | 39 | (29.3) | <0.01 | 48 | (6.4) | 50 | (15.2) | <0.01 |
| Diabetes medication use, % | - | - | - | - | - | - | - | - | - | - | 144 | (72.7) | 115 | (86.5) | <0.01 | - | - | - | - | - |
| Insulin, % | - | - | - | - | - | - | - | - | - | - | 33 | (16.7) | 34 | (25.6) | 0.05 | - | - | - | - | - |
| Metformin, % | - | - | - | - | - | - | - | - | - | - | 131 | (66.2) | 99 | (74.4) | 0.11 | - | - | - | - | - |
| Sulfonylureas, % | - | - | - | - | - | - | - | - | - | - | 35 | (17.7) | 35 | (26.3) | 0.06 | - | - | - | - | - |
| Thiazolidinediones, % | - | - | - | - | - | - | - | - | - | - | 1 | (0.5) | 5 | (3.8) | 0.03 | - | - | - | - | - |
| GLP-1 analogs, % | - | - | - | - | - | - | - | - | - | - | 0 | (0.0) | 4 | (3.0) | 0.01 | - | - | - | - | - |
| DPP-4 inhibitors, % | - | - | - | - | - | - | - | - | - | - | 6 | (3.0) | 2 | (1.5) | 0.38 | - | - | - | - | - |
| Lipid modifying medication use, % | 62 | (14.9) | 29 | (20.4) | 0.12 | 46 | (34.3) | 21 | (38.9) | 0.56 | 151 | (76.3) | 98 | (73.7) | 0.59 | 259 | (34.6) | 148 | (45.0) | <0.01 |
| Data are presented as n (%), mean ± standard deviation or median [interquartile rage]. Abbreviations BMI, body mass index; HDL, high density lipoprotein; LDL, Low density lipoprotein; eGFR, estimated glomerular filtration rate;  Ras, renin-angiotensin system inhibitor; GLP-1, glucagon-like peptide 1; DPP-4, dipeptidyl peptidase-4. All tested with independent sample T-test, Mann Whitney U or Chi-square test as appropriate | | | | | | | | | | | | | | | | | | | | |

| **Supplemental table 2: Clinical characteristics of the study population with TDI echocardiography according to**  **glucose metabolism status (n=748)** | | | | | | |
| --- | --- | --- | --- | --- | --- | --- |
| **Variable** | **NGM (n=416)** | | **Prediabetes (n=134)** | | **T2DM (n=198)** | |
| Demographics |  |  |  |  |  |  |
| Age, years | 57.1 | ±8.1 | 62.1 | ±7.6‡ | 62.8 | ±7.5‡ |
| Women,% | 222 | (53.4) | 51 | (38.1) ‡ | 62 | (31.3) ‡ |
| Measures of (central) obesity |  |  |  |  |  |  |
| BMI, kg/m2 | 25.3 | [23.2-27.5] | 27.1 | [25.4-29.4] ‡ | 28.7 | [25.9-31.6] ‡ |
| Waist circumference, cm |  |  |  |  |  |  |
| Men | 96 | [90-102] | 101 | [96-109] ‡ | 105 | [98-112] ‡ |
| Women | 86 | [79-92] | 93 | [84-99] ‡ | 101 | [89-107] ‡ |
| Waist-to hip ratio^a^ | 0.91 | ±0.08 | 0.96 | ±0.08‡ | 1.00 | ±0.08‡ |
| Blood pressure |  |  |  |  |  |  |
| Office systolic blood pressure, mmHg | 131.0 | ±16.4 | 140.7 | ±17.0‡ | 145.3 | ±18.6‡ |
| Office diastolic blood pressure, mmHg | 75.1 | ±9.8 | 79.6 | ±10.0‡ | 77.9 | ±9.7‡ |
| 24h average ambulatory systolic blood  pressure, mmHg^b^ | 116.5 | ±10.7 | 122.0 | ±12.6‡ | 122.3 | ±11.8‡ |
| 24h average ambulatory diastolic blood  pressure, mmHg^b^ | 73.5 | ±7.0 | 75.3 | ±8.0† | 73.1 | ±7.2 |
| Hypertension, % | 161 | (38.7) | 86 | (64.2) ‡ | 162 | (81.8) ‡ |
| Glucose metabolism |  |  |  |  |  |  |
| Fasting glucose, mmol/L | 5.2 | ±0.4 | 6.0 | ±0.5** | 7.7 | ±2.0** |
| 2h postload glucose^c^ | 5.3 | ±1.1 | 8.2 | ±1.8** | 14.1 | ±3.5** |
| HbA1c, mmol/mol^d^ | 37.0 | ±3.5 | 40.2 | ±4.3** | 50.9 | ±10.4** |
| HbA1c, % ^d^ | 5.5 | ±0.3 | 5.8 | ±0.4** | 6.8 | ±1.0** |
| Lipids |  |  |  |  |  |  |
| Total cholesterol, mmol/L | 5.6 | ±1.0 | 5.5 | ±1.1 | 4.5 | ±1.0‡ |
| HDL cholesterol, mmol/l |  |  |  |  |  |  |
| Men | 1.3 | ±0.3 | 1.3 | ±0.3 | 1.1 | ±0.3‡ |
| Women | 1.7 | ±0.5 | 1.6 | ±0.4 | 1.4 | ±0.4‡ |
| Total to HDL cholesterol ratio | 4.0 | ±1.3 | 4.2 | ±1.3† | 4.0 | ±1.1 |
| LDL cholesterol, mmol/l | 3.5 | ±0.9 | 3.4 | ±1.0 | 2.6 | ±0.9‡ |
| Triglycerides, mmol/l | 1.0 | [0.7-1.4] | 1.4 | [0.9-1.9] ‡ | 1.6 | [1.1-2.1] ‡ |
| Kidney function |  |  |  |  |  |  |
| eGFR, ml/min/1.73 m^2^ | 91.2 | ±13.5 | 85.9 | ±14.0‡ | 85.5 | ±17.0‡ |
| albuminuria, % | 16 | (3.8) | 9 | (6.7) | 34 | (17.2) ‡ |
| Lifestyle |  |  |  |  |  |  |
| Smoking (never, former, current), % | 158/189/69  (38.0/45.4/16.6) | | 41/80/13  (30.6/59.7/9.7)† | | 54/115/29  (27.3/58.1/14.6)† | |
| Alcohol consumption (none, low, high), %^e^ | 54/227/132  (13.1/55.0/32.0) | | 20/66/48  (14.9/49.3/35.8) | | 56/97/44  (28.4/49.2/22.3) ‡ | |
| Moderate to vigorous physical activity, h/wk^f^ | 5.5 | [3.0-8.8] | 4.5 | [2.3-7.5]† | 3.8 | [1.8-6.8] ‡ |
| Prior cardiovascular disease, % | 43 | (10.3) | 20 | (14.9) | 48 | (24.2) ‡ |
| Medication |  |  |  |  |  |  |
| Antihypertensive medication use, % | 89 | (21.4) | 56 | (41.8) ‡ | 131 | (66.2) ‡ |
| Ras inhibitors, % | 60 | (14.4) | 40 | (29.9) ‡ | 106 | (53.5) ‡ |
| Beta- blockers, % | 31 | (7.5) | 29 | (21.6) ‡ | 67 | (33.8) ‡ |
| Diuretics, % | 25 | (6.0) | 24 | (17.9) ‡ | 43 | (21.7) ‡ |
| Calcium antagonists, % | 14 | (3.4) | 8 | (6.0) | 26 | (13.1) ‡ |
| Diabetes medication use, % | - | - | - | - | 144 | (72.7)** |
| Insulin, % | - | - | - | - | 33 | (16.7)** |
| Metformin, % | - | - | - | - | 131 | (66.2)** |
| Sulfonylureas, % | - | - | - | - | 35 | (17.7)** |
| Thiazolidinediones, % | - | - | - | - | 1 | (0.5)** |
| GLP-1 analogs, % | - | - | - | - | 0 | (0.0)** |
| DPP-4 inhibitors, % | - | - | - | - | 6 | (3.0)** |
| Lipid-modifying medication use, % | 62 | (14.9) | 46 | (34.3) ‡ | 151 | (76.3) ‡ |
| Data are presented as n (%), mean ± standard deviation or median [interquartile rage]. Abbreviations: NGM  normal glucose metabolism, pre DM prediabetes, T2DM type 2 diabetes, BMI body mass index, HDL high density  lipoprotein, LDL low density lipoprotein, eGFR estimated glomerular filtration rate, Ras renin-angiotensin system,  GLP-1 glucagon-like peptide-1, DPP-4 dipeptidyl peptidase-4, TDI tissue Doppler imaging. Numbers of missing data:  a n=1, b n=62, c n=43, d n=3, e n=4, f n=108.  P value difference prediabetes or T2DM vs NGM: *<0.10, †<0.05, ‡<0.01, **not applicable | | | | | | |

| **Supplemental table 3a Mediation analyses: Two-dimensional echocardiography (n=714)** | | | | | | |  |  |  |
| --- | --- | --- | --- | --- | --- | --- | --- | --- | --- |
|  |  |  |  |  |  |  |  |  |  |
| A: Associations between independent variable and mediators | | | | | | |  |  |  |
|  | β | P |  | β | P |  |  |  |  |
| - T2DM-LVMI | -0.08 | 0.49 | - prediabetes-LVMI | -0.00 | 0.97 |  |  |  |  |
| - T2DM-E/E’ | 0.16 | 0.12 | - prediabetes-E/E’ | 0.06 | 0.53 |  |  |  |  |
| - T2DM-LVEF | -0.07 | 0.54 | - prediabetes-LVEF | 0.03 | 0.76 |  |  |  |  |
| - T2DM-TR max gr | -0.22 | 0.06 | - prediabetes-TR max gr | -0.33 | <0.01 |  |  |  |  |
|  |  |  |  |  |  |  |  |  |  |
| B: Associations between mediators and dependent variables | | | | | | | | | |
|  | β | P |  | β | P |  | β | P |  |
| - LVMI-RAVI | 0.15 | <0.01 | - LVMI-RV diameter | 0.13 | <0.01 | - LVMI-RV length | 0.10 | <0.01 |  |
| - E/E’-RAVI | -0.07 | 0.07 | - E/E’-RV diameter | -0.02 | 0.64 | - E/E’-RV length | -0.04 | 0.34 |  |
| - LVEF-RAVI | -0.05 | 0.16 | - LVEF-RV diameter | -0.06 | 0.11 | - LVEF-RV length | 0.05 | 0.11 |  |
| - TR max gr-RAVI | 0.11 | <0.01 | - TR max gr-RV diameter | 0.12 | <0.01 | - TR max gr-RV length | -0.07 | 0.04 |  |
|  |  |  |  |  |  |  |  |  |  |
| C: Associations between independent and dependent variables | | | | | | | | | |
|  | β | P |  | β | P |  | β | P |  |
| - T2DM-RAVI total | -0.30 | <0.01 | - T2DM-RV diameter total | -0.42 | <0.01 | - T2DM-RV length total | -0.23 | 0.03 |  |
| - T2DM-RAVI direct | -0.26 | 0.01 | - T2DM-RV diameter direct | -0.38 | <0.01 | - T2DM-RV length direct | -0.23 | 0.03 |  |
|  |  |  |  |  |  |  |  |  |  |
| - prediabetes-RAVI total | -0.29 | <0.01 | - prediabetes-RV diameter total | -0.26 | 0.01 | - prediabetes -RV length total | 0.00 | 0.99 |  |
| - prediabetes-RAVI direct | -0.25 | 0.01 | - prediabetes-RV diameter direct | -0.22 | 0.04 | - prediabetes -RV length direct | -0.02 | 0.84 |  |
|  |  |  |  |  |  |  |  |  |  |
| D: Mediating effects | | | | | | | | | |
|  | ∆β | CI |  | ∆β | CI |  | ∆β | CI |  |
| - T2DM-total-RAVI | -0.04 | (-0.10;0.01) | - T2DM-total-RV diameter | -0.04 | (-0.09;0.01) | - T2DM-total-RV length | 0.00 | (-0.04;0.04) |  |
| - T2DM-LVMI-RAVI | -0.01 | (-0.05;0.02) | - T2DM-LVMI-RV diameter | -0.01 | (-0.05;0.02) | - T2DM-LVMI-RV length | -0.01 | (-0.04;0.01) |  |
| - T2DM-E/E’-RAVI | -0.01 | (-0.04;0.00) | - T2DM-E/E’-RV diameter | 0.00 | (-0.03;0.01) | - T2DM-E/E’-RV length | -0.01 | (-0.03;0.00) |  |
| - T2DM-LVEF-RAVI | 0.00 | (-0.01;0.03) | - T2DM-LVEF-RV diameter | 0.00 | (-0.01;0.03) | - T2DM-LVEF-RV length | 0.00 | (-0.03;0.01) |  |
| - T2DM-TR max gr-RAVI | -0.02 | (-0.06;0.00) | - T2DM-TR max gr-RV diameter | -0.03 | (-0.07;0.00) | - T2DM-TR max gr-RV length | 0.02 | (0.00;0.05) |  |
|  |  |  |  |  |  |  |  |  |  |
| - prediabetes-total-RAVI | -0.04 | (-0.10;0.01) | - prediabetes-total-RV diameter | -0.04 | (-0.10;0.00) | - prediabetes-total-RV length | 0.02 | (-0.02;0.07) |  |
| - prediabetes-LVMI-RAVI | 0.00 | (-0.03;0.04) | - prediabetes-LVMI-RV diameter | 0.00 | (-0.03;0.03) | - prediabetes-LVMI-RV length | 0.00 | (-0.02;0.02) |  |
| - prediabetes-E/E’-RAVI | 0.00 | (-0.03;0.01) | - prediabetes-E/E’-RV diameter | 0.00 | (-0.02;0.00) | - prediabetes-E/E’-RV length | 0.00 | (-0.02;0.00) |  |
| - prediabetes-LVEF-RAVI | 0.00 | (-0.02;0.01) | - prediabetes-LVEF-RV diameter | 0.00 | (-0.02;0.01) | - prediabetes-LVEF-RV length | 0.00 | (-0.01;0.02) |  |
| - prediabetes-TR max gr-RAVI | -0.04 | (-0.08;-0.01) | - prediabetes-TR max gr-RV diameter | -0.04 | (-0.09;-0.01) | - prediabetes-TR max gr-RV length | 0.02 | (0.00;0.06) |  |
|  |  |  |  |  |  |  |  |  |  |
| All analyses were adjusted for age, office systolic blood pressure, antihypertensive medication, smoking status, prior cardiovascular disease,  waist circumference, albuminuria, eGFR, total to high density lipoprotein cholesterol ratio, triglycerides, the use of lipid-modifying medication.  Abbreviations: RV, right ventricle; RA, right atrium; LVMI, left ventricular mass index; E/E’, peak flow velocity E/ longitudinal velocities E ratio;  LVEF, left ventricular ejection fraction; TR max grad, maximum gradient of the tricuspid valve.T2DM type 2 diabetes mellitus. | | | | | | | | | |

| **Supplemental table 3b Mediation analyses: Tissue Doppler imaging echocardiography (n=732)** | | | | | | | | | | | | | | | | | |
| --- | --- | --- | --- | --- | --- | --- | --- | --- | --- | --- | --- | --- | --- | --- | --- | --- | --- |
|  |  | |  | |  |  |  |  |  |  |  |  |  |  |  |  |  |
| A: Associations between independent variable and mediators | | | | | | | | |  |  |  |  |  |  |  |  |  |
|  | | β | | P |  | β | P |  |  |  |  |  |  |  |  |  |  |
| - T2DM-LVMI | | -0.08 | | 0.46 | - preDM-LVMI | 0.00 | 0.98 |  |  |  |  |  |  |  |  |  |  |
| - T2DM-E/E’ | | 0.16 | | 0.13 | - preDM-E/E’ | 0.10 | 0.29 |  |  |  |  |  |  |  |  |  |  |
| - T2DM-LVEF | | -0.09 | | 0.47 | - preDM-LVEF | -0.03 | 0.82 |  |  |  |  |  |  |  |  |  |  |
| - T2DM-TRmaxGr | | -0.23 | | 0.06 | - preDM-TRmaxGr | -0.36 | <0.01 |  |  |  |  |  |  |  |  |  |  |
|  | |  | |  |  |  |  |  |  |  |  |  |  |  |  |  |  |
| B: Associations between mediators and dependent variables | | | | | | | | | | | | | | | | | |
|  | | β | | P |  | β | P |  | β | P |  | β | P |  | β | P |  |
| - LVMI-S’RV | | 0.08 | | 0.04 | - LVMI-E’RV | -0.03 | 0.40 | - LVMI-A’RV | -0.01 | 0.85 | - LVMI- E’/A’RV | -0.03 | 0.39 | - LVMI- MPI | 0.01 | 0.78 |  |
| - E/E’-S’RV | | 0.07 | | 0.07 | - E/E’-E’RV | 0.09 | 0.03 | - E/E’-A’RV | -0.02 | 0.61 | - E/E’- E’/A’RV | 0.07 | 0.04 | - E/E’- MPI | -0.19 | <0.01 |  |
| - LVEF-S’RV | | 0.11 | | <0.01 | - LVEF-E’RV | 0.05 | 0.17 | - LVEF-A’RV | 0.08 | 0.02 | - LVEF- E’/A’RV | -0.04 | 0.16 | - LVEF- MPI | -0.01 | 0.76 |  |
| - TRmaxGr-S’RV | | 0.05 | | 0.12 | - TRmaxGr-E’RV | 0.07 | 0.04 | - TRmaxGr-A’RV | 0.04 | 0.26 | - TRmaxGr- E’/A’RV | 0.02 | 0.45 | - TRmaxGr- MPI | -0.08 | 0.02 |  |
|  | |  | |  |  |  |  |  |  |  |  |  |  |  |  |  |  |
| C: Associations between independent and dependent variables | | | | | | | | | | | | | | | | | |
|  | | β | | P |  | β | P |  | β | P |  | β | P |  | β | P |  |
| - T2DM-S’RV total | | -0.31 | | <0.01 | - T2DM-E’RV total | -0.23 | 0.04 | - T2DM-A’RV total | 0.19 | 0.07 | - T2DM- E’/A’RV total | -0.04 | 0.61 | - T2DM- MPI total | -0.20 | 0.08 |  |
| - T2DM-S’RV direct | | -0.29 | | <0.01 | - T2DM-E’RV direct | -0.23 | 0.04 | - T2DM-A’RV direct | 0.18 | 0.10 | - T2DM- E’/A’RV direct | -0.06 | 0.53 | - T2DM- MPI direct | -0.19 | 0.09 |  |
|  | |  | |  |  |  |  |  |  |  |  |  |  |  |  |  |  |
| - preDM-S’RV total | | -0.18 | | 0.09 | - preDM-E’RV total | -0.26 | 0.01 | - preDM-A’RV total | -0.25 | 0.01 | - preDM-E’/A’RV total | -0.03 | 0.75 | - preDM-MPI total | -0.11 | 0.32 |  |
| - preDM-S’RV direct | | -0.17 | | 0.11 | - preDM-E’RV direct | -0.24 | 0.02 | - preDM-A’RV direct | -0.23 | 0.02 | - preDM-E’/A’RV direct | -0.03 | 0.75 | - preDM-MPI direct | -0.12 | 0.27 |  |
|  | |  | |  |  |  |  |  |  |  |  |  |  |  |  |  |  |
| D: Mediating effects | | | | | | | | | | | | | | | | | |
|  | | ∆β | | CI |  | ∆β | CI |  | ∆β | CI |  | ∆β | CI |  | ∆β | CI |  |
| - T2DM-total-S’RV | | -0.02 | | (-0.07;0.03) | - T2DM-total-E’RV | 0.00 | (-0.04;0.04) | - T2DM-total-A’RV | -0.02 | (-0.06;0.01) | - T2DM-total-E’/A’RV | 0.01 | (-0.01;0.05) | - T2DM-total-MPI | -0.01 | (-0.07;0.04) |  |
| - T2DM-LVMI-S’RV | | -0.01 | | (-0.04;0.01) | - T2DM-LVMI-E’RV | 0.00 | (0.00;0.02) | - T2DM-LVMI-A’RV | 0.00 | (-0.01;0.02) | - T2DM-LVMI-E’/A’RV | 0.00 | (0.00;0.02) | - T2DM-LVMI-MPI | 0.00 | (-0.02;0.01) |  |
| - T2DM-E/E’-S’RV | | 0.01 | | (0.00;0.04) | - T2DM-E/E’-E’RV | 0.01 | (0.00;0.05) | - T2DM-E/E’-A’RV | 0.00 | (-0.03;0.01) | - T2DM-E/E’-E’/A’RV | 0.01 | (0.00;0.04) | - T2DM-E/E’-MPI | -0.03 | (-0.08;0.00) |  |
| - T2DM-LVEF-S’RV | | -0.01 | | (-0.05;0.02) | - T2DM-LVEF-E’RV | 0.00 | (-0.03;0.01) | - T2DM-LVEF-A’RV | -0.01 | (-0.04;0.12) | - T2DM-LVEF-E’/A’RV | 0.00 | (-0.01;0.03) | - T2DM-LVEF-MPI | 0.00 | (-0.01;0.02) |  |
| - T2DM-TRmaxGr-S’RV | | -0.01 | | (-0.05;0.00) | - T2DM-TRmaxGr-E’RV | -0.02 | (-0.05;0.00) | - T2DM-TRmaxGr-A’RV | -0.01 | (-0.04;0.00) | - T2DM-TRmaxGr-E’/A’RV | -0.01 | (-0.03;0.01) | - T2DM-TRmaxGr-MPI | 0.02 | (0.00;0.06) |  |
|  | |  | |  |  |  |  |  |  |  |  |  |  |  |  |  |  |
| - preDM-total-S’RV | | -0.01 | | (-0.06;0.03) | - preDM-total-E’RV | -0.02 | (-0.06;0.02) | - preDM-total-A’RV | -0.02 | (-0.06;0.01) | - preDM-total-E’/A’RV | 0.00 | (-0.03;0.03) | - preDM-total-MPI | 0.01 | (-0.04;0.06) |  |
| - preDM-LVMI-S’RV | | 0.00 | | (-0.02;0.02) | - preDM-LVMI-E’RV | 0.00 | (-0.01;0.01) | - preDM-LVMI-A’RV | 0.00 | (-0.01;0.01) | - preDM-LVMI-E’/A’RV | 0.00 | (-0.01;0.01) | - preDM-LVMI-MPI | 0.00 | (-0.01;0.01) |  |
| - preDM-E/E’-S’RV | | 0.01 | | (0.00;0.03) | - preDM-E/E’-E’RV | 0.01 | (0.00;0.04) | - preDM-E/E’-A’RV | 0.00 | (-0.02;0.01) | - preDM-E/E’-E’/A’RV | 0.01 | (0.00;0.03) | - preDM-E/E’-E’/A’RV | -0.02 | (-0.06;0.01) |  |
| - preDM-LVEF-S’RV | | 0.00 | | (-0.03;0.02) | - preDM-LVEF-E’RV | 0.00 | (-0.02;0.01) | - preDM-LVEF-A’RV | 0.00 | (-0.03;0.01) | - preDM-LVEF-E’/A’RV | 0.00 | (-0.01;0.02) | - preDM-LVEF-E’/A’RV | 0.00 | (-0.01;0.01) |  |
| - preDM-TRmaxGr-S’RV | | -0.02 | | (-0.06;0.00) | - preDM-TRmaxGr-E’RV | -0.02 | (-0.07;0.00) | - preDM-TRmaxGr-A’RV | -0.01 | (-0.05;0.01) | - preDM-TRmaxGr-E’/A’RV | -0.01 | (-0.04;0.01) | - preDM-TRmaxGr-E’/A’RV | 0.03 | (0.01;0.07) |  |
|  | |  | |  |  |  |  |  |  |  |  |  |  |  |  |  |  |
| All analyses were adjusted for age, office systolic blood pressure, antihypertensive medication, smoking status, prior cardiovascular disease, waist circumference, albuminuria, eGFR, total to high density lipoprotein cholesterol ratio, triglycerides, the use of lipid-modifying medication. Abbreviations: T2DM, type 2 diabetes mellitus; preDM, prediabetes; RV, right ventricle; RA, right atrium; LVMI, left ventricular mass index; E/E’, peak flow velocity E/ longitudinal velocities E ratio; LVEF, left ventricular ejection fraction; TRmaxGr, maximum gradient of the tricuspid valve. | | | | | | | | | | | | | | | | | |

| **Supplemental table 4a: Multivariable adjusted differences in right ventricle structure and function between**  **individuals with normal glucose metabolism, prediabetes and type 2 diabetes (Population with full**  **echocardiographic data and mediators)** | | | | | |
| --- | --- | --- | --- | --- | --- |
| **Variable** | **Model** | **NGM** | **Pre-diabetes** | **T2DM** |  |
|  |  |  | **B (95% CI)** | **B (95% CI)** | **P for trend** |
| RA volume index (SD)^a^ | 1 | Ref | -0.43 (-0.64;-0.22) ‡ | -0.59 (-0.77;-0.40) ‡ | <0.01 |
|  | 2 | Ref | -0.37 (-0.59;-0.16) ‡ | -0.49 (-0.69;-0.28) ‡ | <0.01 |
|  | 3 | Ref | -0.32 (-0.54;-0.11) ‡ | -0.40 (-0.63;-0.18) ‡ | <0.01 |
|  |  |  |  |  |  |
| RV diameter (SD)^a^ | 1 | Ref | -0.09 (-0.31;0.13) | -0.23 (-0.42;-0.04) † | 0.02 |
|  | 2 | Ref | -0.24 (-0.46;-0.01) † | -0.47 (-0.68;-0.26) ‡ | <0.01 |
|  | 3 | Ref | -0.21 (-0.44;0.01) * | -0.44 (-0.67;-0.20) ‡ | <0.01 |
|  |  |  |  |  |  |
| RV length (SD)^a^ | 1 | Ref | 0.23 (0.02;0.44) † | 0.10 (-0.08;0.28) | 0.19 |
|  | 2 | Ref | 0.09 (-0.13;0.30) | -0.16 (-0.36;0.04) | 0.16 |
|  | 3 | Ref | 0.07 (-0.14;0.29) | -0.25 (-0.47;-0.03) † | 0.05 |
|  |  |  |  |  |  |
| S' RV (SD)^a^ | 1 | Ref | -0.17 (-0.39;0.05) | -0.23 (-0.42;-0.04) † | 0.01 |
|  | 2 | Ref | -0.21 (-0.43;0.01) * | -0.26 (-0.48;-0.05) † | 0.01 |
|  | 3 | Ref | -0.21 (-0.43;0.02) * | -0.28 (-0.51;-0.05) † | 0.01 |
|  |  |  |  |  |  |
| TDI E' RV (SD)^a^ | 1 | Ref | -0.35 (-0.56;-0.14) ‡ | -0.32 (-0.50;-0.14) ‡ | <0.01 |
|  | 2 | Ref | -0.36 (-0.58;-0.15) ‡ | -0.31 (-0.52;-0.11) ‡ | <0.01 |
|  | 3 | Ref | -0.35 (-0.56;-0.13) ‡ | -0.30 (-0.53;-0.07) † | <0.01 |
|  |  |  |  |  |  |
| TDI A' RV (SD)^a^ | 1 | Ref | -0.29 (-0.50;-0.08) ‡ | -0.29 (-0.47;-0.10) ‡ | <0.01 |
|  | 2 | Ref | -0.26 (-0.48;-0.05) † | -0.20 (-0.40;0.01) * | 0.04 |
|  | 3 | Ref | -0.24 (-0.46;-0.02) † | -0.18 (-0.41;0.04) | 0.07 |
|  |  |  |  |  |  |
| TDI E’/A’ ratio (SD)^a^ | 1 | Ref | -0.07 (-0.26;0.12) | -0.06 (-0.22;0.11) | 0.47 |
|  | 2 | Ref | -0.10 (-0.29;0.10) | -0.13 (-0.31;0.06) | 0.17 |
|  | 3 | Ref | -0.10 (-0.30;0.09) | -0.14 (-0.34;0.07) | 0.17 |
|  |  |  |  |  |  |
| Myocardial performance index RV (SD)^a^ | 1 | Ref | -0.13 (-0.34;0.08) | -0.10 (-0.28;0.08) | 0.24 |
|  | 2 | Ref | -0.12 (-0.34;0.10) | -0.09 (-0.30;0.11) | 0.34 |
|  | 3 | Ref | -0.17 (-0.39;0.05) | -0.20 (-0.42;0.03) * | 0.07 |
| Model 1: adjusted for age. Model 2: adjusted for model 1+ office systolic blood pressure, antihypertensive medication,  smoking status, prior cardiovascular disease, waist circumference. Model 3: adjusted for model 2+ albuminuria , eGFR,  total to high density lipoprotein cholesterol ratio, triglycerides, the use of lipid-modifying medication.  Abbreviations: CI confidence interval, MPI myocardial performance index , NGM normal glucose metabolism, RA right  atrial, RV right ventricular, SD standard deviation, T2DM type 2 diabetes mellitus, TDI tissue Doppler imaging.  Study population a n=696. P value difference prediabetes or T2DM vs NGM: *<0.10, †<0.05, ‡<0.01 | | | | | |

| **Supplemental table 4b: Multivariable adjusted differences in right ventricle structure and function between**  **individuals with normal glucose metabolism, prediabetes and type 2 diabetes (24 hour ambulatory systolic**  **bloodpressure in stead of office systolic blood pressure)** | | | | | |
| --- | --- | --- | --- | --- | --- |
| **Variable** | **Model** | **NGM** | **Prediabetes** | **T2DM** |  |
|  |  |  | **B (95% CI)** | **B (95% CI)** | **P for trend** |
| RA volume index (SD)^a^ | 1 | Ref | -0.40 (-0.59;-0.21) ‡ | -0.54 (-0.70;-0.38) ‡ | <0.01 |
|  | 2 | Ref | -0.34 (-0.54;-0.15) ‡ | -0.45 (-0.63;-0.26) ‡ | <0.01 |
|  | 3 | Ref | -0.29 (-0.48;-0.09) ‡ | -0.34 (-0.54;-0.14) ‡ | <0.01 |
|  |  |  |  |  |  |
| RV diameter (SD)^a^ | 1 | Ref | -0.10 (-0.31;0.10) | -0.21 (-0.39;-0.04) † | 0.02 |
|  | 2 | Ref | -0.26 (-0.46;-0.05) † | -0.50 (-0.70;-0.31) ‡ | <0.01 |
|  | 3 | Ref | -0.24 (-0.45;-0.03) † | -0.47 (-0.68;-0.25) ‡ | <0.01 |
|  |  |  |  |  |  |
| RV length (SD)^a^ | 1 | Ref | 0.20 (0.00;0.40) | 0.14 (-0.03;0.31) | 0.08 |
|  | 2 | Ref | 0.04 (-0.17;0.24) | -0.15 (-0.34;0.04) † | 0.15 |
|  | 3 | Ref | 0.03 (-0.18;0.23) | -0.23 (-0.43;-0.02) † | 0.05 |
|  |  |  |  |  |  |
| S' RV (SD)^b^ | 1 | Ref | -0.14 (-0.35;0.07) | -0.25 (-0.44;-0.07) ‡ | 0.01 |
|  | 2 | Ref | -0.18 (-0.40;0.03) * | -0.28 (-0.49;-0.07) | 0.01 |
|  | 3 | Ref | -0.17 (-0.38;0.05) | -0.27 (-0.50;-0.04) | 0.02 |
|  |  |  |  |  |  |
| TDI E' RV (SD)^b^ | 1 | Ref | -0.28 (-0.48;-0.07) ‡ | -0.35 (-0.53;-0.17) ‡ | <0.01 |
|  | 2 | Ref | -0.28 (-0.49;-0.07) † | -0.33 (-0.53;-0.12) ‡ | <0.01 |
|  | 3 | Ref | -0.24 (-0.45;-0.03) † | -0.28 (-0.51;-0.06) † | 0.01 |
|  |  |  |  |  |  |
| TDI A' RV (SD)^b^ | 1 | Ref | -0.28 (-0.48;-0.08) ‡ | -0.31 (-0.48;-0.13) ‡ | <0.01 |
|  | 2 | Ref | -0.25 (-0.46;-0.05) † | -0.22 (-0.42;-0.02) † | 0.02 |
|  | 3 | Ref | -0.22 (-0.42;-0.01) † | -0.18 (-0.39;0.04) | 0.07 |
|  |  |  |  |  |  |
| TDI E’/A’ ratio (SD)^b^ | 1 | Ref | -0.01 (-0.19;0.16) | -0.05 (-0.21;0.10) | 0.50 |
|  | 2 | Ref | -0.03 (-0.22;0.15) | -0.11 (-0.29;0.06) | 0.22 |
|  | 3 | Ref | -0.03 (-0.21;0.16) | -0.12 (-0.31;0.08) | 0.26 |
|  |  |  |  |  |  |
| Myocardial performance index RV (SD)^b^ | 1 | Ref | -0.09 (-0.29;0.12) | -0.07 (-0.25;0.11) | 0.40 |
|  | 2 | Ref | -0.08 (-0.29;0.13) | -0.08 (-0.28;0.13) | 0.44 |
|  | 3 | Ref | -0.13 (-0.34;0.08) | -0.17 (-0.39;0.06) | 0.12 |
| Model 1: adjusted for age. Model 2: adjusted for model 1+ 24 hour ambulatory blood pressure, antihypertensive  medication, smoking status, prior cardiovascular disease, waist circumference. Model 3: adjusted for model 2+  albuminuria, eGFR, total to high density lipoprotein cholesterol ratio, triglycerides, the use of lipid-modifying  medication.  Abbreviations: CI confidence interval, MPI myocardial performance index , NGM normal glucose metabolism,  RA right atrial, RV right ventricular, SD standard deviation, T2DM type 2 diabetes mellitus, TDI tissue Doppler imaging.  Study population a n=723 b n=686. P value difference prediabetes or T2DM vs NGM: *<0.10, †<0.05, ‡<0.01 | | | | | |

| **Supplemental table 4c: Multivariable adjusted differences in right ventricle structure and function between**  **individuals with normal glucose metabolism, prediabetes and type 2 diabetes ( additionally adjusted for renin**  **angiotensin system modifying agents)** | | | | | |
| --- | --- | --- | --- | --- | --- |
| **Variable** | **Model** | **NGM** | **Prediabetes** | **T2DM** |  |
|  |  |  | **B (95% CI)** | **B (95% CI)** | **P for trend** |
| RA volume index (SD)^a^ | 1 | Ref | -0.39 (-0.58;-0.21) ‡ | -0.52 (-0.68;-0.36) ‡ | <0.01 |
|  | 2 | Ref | -0.32 (-0.51;-0.13) ‡ | -0.39 (-0.57;-0.21) ‡ | <0.01 |
|  | 3 | Ref | -0.26 (-0.45;-0.07) ‡ | -0.29 (-0.48;-0.09) ‡ | <0.01 |
|  | 4 | Ref | -0.26 (-0.45;-0.07) ‡ | -0.29 (-0.49;-0.10) ‡ | <0.01 |
|  |  |  |  |  |  |
| RV diameter (SD)^a^ | 1 | Ref | -0.12 (-0.31;0.08) | -0.20 (-0.37;-0.04) † | 0.02 |
|  | 2 | Ref | -0.29 (-0.49;-0.09) ‡ | -0.49 (-0.68;-0.30) ‡ | <0.01 |
|  | 3 | Ref | -0.27 (-0.47;-0.07) ‡ | -0.44 (-0.65;-0.24) ‡ | <0.01 |
|  | 4 | Ref | -0.27 (-0.47;-0.07) ‡ | -0.45 (-0.65;-0.24) ‡ | <0.01 |
|  |  |  |  |  |  |
| RV length (SD)^a^ | 1 | Ref | -0.15 (-0.04;0.34) | -0.15 (-0.01;-0.32) * | 0.06 |
|  | 2 | Ref | -0.02 (-0.22;0.17) | -0.15 (-0.33;0.04) | 0.13 |
|  | 3 | Ref | -0.04 (-0.24;0.15) | -0.22 (-0.42;-0.02) † | 0.04 |
|  | 4 | Ref | -0.04 (-0.24;0.15) | -0.22 (-0.42;-0.02) † | 0.04 |
|  |  |  |  |  |  |
| S' RV (SD)^b^ | 1 | Ref | -0.18 (-0.37;0.02) * | -0.26 (-0.44;-0.09) ‡ | <0.01 |
|  | 2 | Ref | -0.21 (-0.41;0.00) † | -0.30 (-0.50;-0.10) ‡ | <0.01 |
|  | 3 | Ref | -0.19 (-0.39;0.02) * | -0.29 (-0.51;-0.07) ‡ | 0.01 |
|  | 4 | Ref | -0.19 (-0.39;0.02) * | -0.30 (-0.52;-0.08) ‡ | 0.01 |
|  |  |  |  |  |  |
| TDI E' RV (SD)^b^ | 1 | Ref | -0.31 (-0.50;-0.11) ‡ | -0.36 (-0.53;-0.18) ‡ | <0.01 |
|  | 2 | Ref | -0.30 (-0.50;-0.10) ‡ | -0.31 (-0.51;-0.11) ‡ | <0.01 |
|  | 3 | Ref | -0.26 (-0.47;-0.06) † | -0.26 (-0.48;-0.05) † | 0.01 |
|  | 4 | Ref | -0.26 (-0.47;-0.06) † | -0.27 (-0.45;-0.06) † | 0.01 |
|  |  |  |  |  |  |
| TDI A' RV (SD)^b^ | 1 | Ref | -0.32 (-0.51;-0.13) ‡ | -0.29 (-0.46;-0.12) ‡ | <0.01 |
|  | 2 | Ref | -0.29 (-0.49;-0.10) ‡ | -0.23 (-0.42;-0.03) † | 0.01 |
|  | 3 | Ref | -0.26 (-0.46;-0.07) ‡ | -0.20 (-0.41;0.01) * | 0.04 |
|  | 4 | Ref | -0.26 (-0.46;-0.07) ‡ | -0.20 (-0.41;0.01) * | 0.04 |
|  |  |  |  |  |  |
| TDI E’/A’ ratio (SD)^b^ | 1 | Ref | -0.02 (-0.19;0.15) | -0.07 (-0.22;0.08) | 0.39 |
|  | 2 | Ref | -0.03 (-0.20;0.15) | -0.08 (-0.25;0.09) | 0.35 |
|  | 3 | Ref | -0.02 (-0.19;0.16) | -0.07 (-0.25;0.12) | 0.50 |
|  | 4 | Ref | -0.02 (-0.19;0.16) | -0.07 (-0.26;0.12) | 0.46 |
|  |  |  |  |  |  |
| Myocardial performance index RV (SD)^b^ | 1 | Ref | -0.04 (-0.23;0.16) | -0.04 (-0.21;0.13) | 0.63 |
|  | 2 | Ref | -0.06 (-0.26;0.15) | -0.09 (-0.29;0.12) | 0.40 |
|  | 3 | Ref | -0.10 (-0.31;0.11) | -0.17 (-0.39;0.05) | 0.12 |
|  | 4 | Ref | -0.10 (-0.31;0.10) | -0.18 (-0.40;0.04) | 0.10 |
| Model 1: adjusted for age. Model 2: adjusted for model 1+ office systolic blood pressure, antihypertensive medication,  smoking status, prior cardiovascular disease, waist circumference. Model 3: adjusted for model 2+ albuminuria, eGFR,  total to high density lipoprotein cholesterol ratio, triglycerides, the use of lipid-modifying medication. Model 4:  adjusted for model 3 + renin angiotensin system modifying agents.  Abbreviations: CI confidence interval, MPI myocardial performance index , NGM normal glucose metabolism,  RA right atrial, RV right ventricular, SD standard deviation, T2DM type 2 diabetes mellitus, TDI tissue Doppler imaging.  Study population a n=792 b n=748. P value difference prediabetes or T2DM vs NGM: *<0.10, †<0.05, ‡<0.01 | | | | | |

| **Supplemental table 4d: Multivariable adjusted differences in right ventricle structure and function between**  **individuals with normal glucose metabolism, prediabetes and type 2 diabetes (additionally adjusted for**  **moderate to vigorous physical activity )** | | | | | |
| --- | --- | --- | --- | --- | --- |
| **Variable** | **Model** | **NGM** | **Prediabetes** | **T2DM** |  |
|  |  |  | **B (95% CI)** | **B (95% CI)** | **P for trend** |
| RA volume index (SD)^a^ | 1 | Ref | -0.41 (-0.61;-0.21) ‡ | -0.44 (-0.62;-0.27) ‡ | <0.01 |
|  | 2 | Ref | -0.32 (-0.52;-0.11) ‡ | -0.30 (-0.50;-0.10) ‡ | <0.01 |
|  | 3 | Ref | -0.26 (-0.47;-0.06) † | -0.22 (-0.43;-0.01) † | 0.03 |
|  | 4 | Ref | -0.26 (-0.47;-0.06) † | -0.22 (-0.43;0.00) * | 0.03 |
|  |  |  |  |  |  |
| RV diameter (SD)^a^ | 1 | Ref | -0.14 (-0.34;0.07) | -0.21 (-0.40;-0.03) † | 0.02 |
|  | 2 | Ref | -0.30 (-0.52;-0.09) ‡ | -0.49 (-0.70;-0.28) ‡ | <0.01 |
|  | 3 | Ref | -0.28 (-0.50;-0.06) † | -0.45 (-0.68;-0.23) ‡ | <0.01 |
|  | 4 | Ref | -0.27 (-0.48;-0.05) † | -0.42 (-0.65;-0.20) ‡ | <0.01 |
|  |  |  |  |  |  |
| RV length (SD)^a^ | 1 | Ref | 0.22 (0.02;0.42) † | 0.19 (0.01;0.37) † | 0.02 |
|  | 2 | Ref | 0.06 (-0.15;0.26) | -0.09 (-0.30;0.11) | 0.41 |
|  | 3 | Ref | 0.04 (-0.17;0.24) | -0.18 (-0.40;0.03) * | 0.12 |
|  | 4 | Ref | 0.04 (-0.17;0.25) | -0.18 (-0.40;0.04) | 0.14 |
|  |  |  |  |  |  |
| S' RV (SD)^b^ | 1 | Ref | -0.21 (-0.42;0.00) † | -0.21 (-0.40;-0.02) † | 0.02 |
|  | 2 | Ref | -0.23 (-0.45;-0.01) † | -0.22 (-0.44;0.00) * | 0.04 |
|  | 3 | Ref | -0.21 (-0.43;0.01) * | -0.24 (-0.48;-0.01) † | 0.03 |
|  | 4 | Ref | -0.20 (-0.42;0.02) * | -0.22 (-0.46;0.02) * | 0.05 |
|  |  |  |  |  |  |
| TDI E' RV (SD)^b^ | 1 | Ref | -0.30 (-0.51;-0.09) ‡ | -0.26 (-0.45;-0.07) ‡ | <0.01 |
|  | 2 | Ref | -0.28 (-0.50;-0.06) † | -0.20 (-0.42;0.02) * | 0.04 |
|  | 3 | Ref | -0.25 (-0.46;-0.03) † | -0.18 (-0.42;0.05) | 0.08 |
|  | 4 | Ref | -0.24 (-0.46;-0.02) † | -0.16 (-0.70;0.08) | 0.12 |
|  |  |  |  |  |  |
| TDI A' RV (SD)^b^ | 1 | Ref | -0.33 (-0.54;-0.13) ‡ | -0.23 (-0.42;-0.05) † | 0.01 |
|  | 2 | Ref | -0.31 (-0.52;-0.1) ‡ | -0.17 (-0.38;0.04) | 0.07 |
|  | 3 | Ref | -0.29 (-0.5;-0.07) ‡ | -0.15 (-0.38;0.08) | 0.11 |
|  | 4 | Ref | -0.28 (-0.5;-0.07) ‡ | -0.15 (-0.38;0.08) | 0.12 |
|  |  |  |  |  |  |
| TDI E’/A’ ratio (SD)^b^ | 1 | Ref | 0.00 (-0.18;0.18) | -0.04 (-0.21;0.12) | 0.63 |
|  | 2 | Ref | 0.01 (-0.18;0.20) | -0.05 (-0.24;0.14) | 0.66 |
|  | 3 | Ref | 0.02 (-0.17;0.21) | -0.04 (-0.24;0.17) | 0.77 |
|  | 4 | Ref | 0.03 (-0.17;0.22) | -0.02 (-0.23;0.19) | 0.88 |
|  |  |  |  |  |  |
| Myocardial performance index RV (SD)^b^ | 1 | Ref | -0.04 (-0.26;0.17) | -0.02 (-0.22;0.17) | 0.78 |
|  | 2 | Ref | -0.07 (-0.29;0.16) | -0.06 (-0.29;0.16) | 0.54 |
|  | 3 | Ref | -0.11 (-0.33;0.11) | -0.14 (-0.38;0.10) | 0.22 |
|  | 4 | Ref | -0.12 (-0.34;0.11) | -0.17 (-0.41;0.07) | 0.15 |
| Model 1: adjusted for age. Model 2: adjusted for model 1+ office systolic blood pressure, antihypertensive medication,  smoking status, prior cardiovascular disease, waist circumference. Model 3: adjusted for model 2+ albuminuria, eGFR,  total to high density lipoprotein cholesterol ratio, triglycerides, the use of lipid-modifying medication. Model 4:  adjusted for model 3 + moderate to vigorous physical activity.  Abbreviations: CI confidence interval, MPI myocardial performance index , NGM normal glucose metabolism,  RA right atrial, RV right ventricular, SD standard deviation, T2DM type 2 diabetes mellitus, TDI tissue Doppler imaging.  Study population a n=684 b n=640. P value difference prediabetes or T2DM vs NGM: *<0.10, †<0.05, ‡<0.01 | | | | | |

| **Supplemental table 4e: Multivariable adjusted differences in right ventricle structure and function between**  **individuals with normal glucose metabolism, prediabetes and type 2 diabetes (population without atrial**  **fibrillation, wall motion abnormalities, significant valvular pathology, and prior cardiovascular disease)** | | | | | |
| --- | --- | --- | --- | --- | --- |
| **Variable** | **Model** | **NGM** | **Prediabetes** | **T2DM** |  |
|  |  |  | **B (95% CI)** | **B (95% CI)** | **P for trend** |
| RA volume index (SD)^a^ | 1 | Ref | -0.34 (0.55;-0.13) ‡ | -0.56 (-0.75;-0.37) ‡ | <0.01 |
|  | 2 | Ref | -0.25 (-0.47;-0.04) † | -0.41 (-0.63;-0.20) ‡ | <0.01 |
|  | 3 | Ref | -0.21 (-0.42;0.01) * | -0.32 (-0.55;-0.09) ‡ | 0.01 |
|  |  |  |  |  |  |
| RV diameter (SD)^a^ | 1 | Ref | -0.12 (-0.34;0.09) | -0.24 (-0.44;-0.05) † | 0.02 |
|  | 2 | Ref | -0.28 (-0.50;-0.05) † | -0.47 (-0.69;-0.26) ‡ | <0.01 |
|  | 3 | Ref | -0.27 (-0.49;-0.04) † | -0.44 (-0.68;-0.20) ‡ | <0.01 |
|  |  |  |  |  |  |
| RV length (SD)^a^ | 1 | Ref | 0.12 (-0.09;0.33) | 0.12 (-0.07;0.31) | 0.20 |
|  | 2 | Ref | -0.05 (-0.26;0.16) | -0.15 (-0.36;0.06) | 0.15 |
|  | 3 | Ref | -0.07 (-0.28;0.15) | -0.25 (-0.48;-0.02) † | 0.04 |
|  |  |  |  |  |  |
| S' RV (SD)^b^ | 1 | Ref | -0.29 (-0.51;-0.07) † | -0.28 (-0.48;-0.08) ‡ | <0.01 |
|  | 2 | Ref | -0.33 (-0.56;-0.10) ‡ | -0.35 (-0.58;-0.13) ‡ | <0.01 |
|  | 3 | Ref | -0.31 (-0.54;-0.09) ‡ | -0.38 (-0.63;-0.14) ‡ | <0.01 |
|  |  |  |  |  |  |
| TDI E' RV (SD)^b^ | 1 | Ref | -0.38 (-0.59;-0.16) ‡ | -0.40 (-0.60;-0.21) ‡ | <0.01 |
|  | 2 | Ref | -0.37 (-0.59;-0.15) ‡ | -0.36 (-0.58;-0.14) ‡ | <0.01 |
|  | 3 | Ref | -0.34 (-0.56;-0.12) ‡ | -0.34 (-0.58;-0.10) ‡ | <0.01 |
|  |  |  |  |  |  |
| TDI A' RV (SD)^b^ | 1 | Ref | -0.30 (-0.51;-0.09) ‡ | -0.33 (-0.53;-0.14) ‡ | <0.01 |
|  | 2 | Ref | -0.28 (-0.50;-0.07) † | -0.30 (-0.52;-0.09) ‡ | <0.01 |
|  | 3 | Ref | -0.26 (-0.48;-0.04) † | -0.33 (-0.56;-0.09) ‡ | <0.01 |
|  |  |  |  |  |  |
| TDI E’/A’ ratio (SD)^b^ | 1 | Ref | -0.07 (-0.26;0.12) | -0.05 (-0.22;0.12) | 0.50 |
|  | 2 | Ref | -0.07 (-0.26;0.12) | -0.03 (-0.22;0.17) | 0.73 |
|  | 3 | Ref | -0.06 (-0.25;0.14) | 0.01 (-0.20;0.22) | 1.00 |
|  |  |  |  |  |  |
| Myocardial performance index RV (SD)^b^ | 1 | Ref | -0.06 (-0.27;0.16) | -0.07 (-0.26;0.13) | 0.48 |
|  | 2 | Ref | -0.06 (-0.29;0.16) | -0.08 (-0.31;0.14) | 0.44 |
|  | 3 | Ref | -0.09 (-0.31;0.14) | -0.17 (-0.41;0.08) | 0.17 |
| Model 1: adjusted for age. Model 2: adjusted for model 1+ office systolic blood pressure, antihypertensive medication,  smoking status, waist circumference. Model 3: adjusted for model 2+ albuminuria , eGFR, total to high density  lipoprotein cholesterol ratio, triglycerides, the use of lipid-modifying medication.  Abbreviations: CI confidence interval, MPI myocardial performance index , NGM normal glucose metabolism, RA right  atrial, RV right ventricular, SD standard deviation, T2DM type 2 diabetes mellitus, TDI tissue Doppler imaging.  Study population a n=611 b n=597. P value difference prediabetes or T2DM vs NGM: *<0.10, †<0.05, ‡<0.01 | | | | | |
